# Benchmark evaluation of large language models for clinical decision support in headache management

**DOI:** 10.22514/jofph.2026.029

**Published:** 2026-03-12

**Authors:** Shi Chen, Dong Liang, Xu Qiu, Chengqi Dong, Jiayi Deng, Li Xu, Xiaoxue Dong, Yonglei Zhao, Xuemei Fan, Xiaoyu Liu, Yali Wu, Jianliang Sun, Feifang He, Ke Ma, Liang Yu, Hanbin Wang

**Affiliations:** ^1^Department of Pain, Affiliated Hangzhou First People’s Hospital, School of Medicine, Westlake University, 310006 Hangzhou, Zhejiang, China; ^2^The Fourth Clinical School of Medicine, Hangzhou First People’s Hospital, Zhejiang Chinese Medical University, 310006 Hangzhou, Zhejiang, China; ^3^Department of Pain, Wuxi Xishan People’s Hospital, 214000 Wuxi, Jiangsu, China; ^4^National Neuroscience Institute of Singapore, 308433 Singapore, Singapore; ^5^Department of Neurology, Shanghai General Hospital, School of Medicine, Shanghai Jiao Tong University, 200080 Shanghai, China; ^6^Department of Radiology, Sir Run Run Shaw Hospital, School of Medicine, Zhejiang University, 310016 Hangzhou, Zhejiang, China; ^7^Department of Neurology, Affiliated Hangzhou First People’s Hospital, School of Medicine, Westlake University, 310006 Hangzhou, Zhejiang, China; ^8^Affiliated Mental Health Center & Hangzhou Seventh People’s Hospital, School of Medicine, Zhejiang University, 310016 Hangzhou, Zhejiang, China; ^9^Department of Pain Management, Center for Intracranial Hypotension Management, Sir Run Run Shaw Hospital, School of Medicine, Zhejiang University, 310016 Hangzhou, Zhejiang, China; ^10^Department of Pain, Shanghai Ninth People’s Hospital, School of Medicine, Shanghai Jiao Tong University, 200011 Shanghai, China

**Keywords:** Headache disorders, Large language models, Clinical reasoning, Artificial intelligence

## Abstract

**Background**: Headache disorders are a major cause of disability worldwide. In routine practice, diagnosis and guideline-based management are difficult because symptoms can overlap between primary and secondary headaches, and clinicians must combine clinical, imaging, and pathological information. Large language models (LLMs) are being proposed to assist clinical reasoning, but their performance on headache cases and their sensitivity to prompting have not been systematically assessed. **Methods**: We evaluated seven leading LLMs using 13 headache cases from the New England Journal of Medicine (NEJM). We compared two prompting strategies: ask-in-sequence (AS) and ask-at-once (AO). Using a 5-point Likert rubric, three headache specialists independently scored six dimensions: rationality of diagnostic thinking, comprehensiveness of differential diagnosis, diagnostic accuracy, completeness of pathological diagnosis, clinical management, and supplementary value. Readability was measured with Flesch Reading Ease (FRE) and Flesch-Kincaid Grade Level (FKGL). We analyzed differences across models, prompting strategies, and cases. **Results**: Diagnostic accuracy differed by model: in the AS strategy, ChatGPT-4o outperformed Grok-3. Supplementary value also varied: in AS, Grok-3 outperformed ChatGPT-5 and Hunyuan-T1; in AO, DeepSeek-R1 outperformed ChatGPT-5. Overall, supplementary value was generally higher with AS, while strategy-related differences in diagnostic accuracy were observed only for Grok-3. Performance also depended on the case; C8 and C11 consistently received very low scores, suggesting difficulty integrating psychiatric or warning signs with pathological findings. Readability differed significantly: Gemini 2.5 Pro had the highest FRE (best readability) across strategies, and AS outputs generally had higher FRE. Within AS, ChatGPT-4o had the highest FKGL (worst readability). No significant model differences were found for the other four clinical dimensions. **Conclusions**: This study provides a structured, reproducible evaluation of LLMs on headache case analysis. While some models improved supplementary value, diagnostic accuracy, or readability, overall clinical accuracy remains below expert performance and is not sufficient for unsupervised clinical use.

## 1. Introduction

Headache disorders represent a significant global health burden, affecting over 50% of adults annually and accounting for approximately 5.4% of all years lived with disability [1, 2, 3, 4]. The third edition of the International Classification of Headache Disorders (ICHD-3) is the primary standard for diagnosing and classifying headaches; yet diagnostic errors remain common in primary care settings. Surveys indicate persistent under-identification and misclassification, as symptoms often overlap among primary headache disorders, such as migraine and tension-type headache, and secondary causes, including intracranial hemorrhage or central nervous system inflammation [5, 6]. Clinical management is further complicated by the interpretation of subtle patient histories and relating symptoms to neuroimaging/genetic information, as well as polypharmacy risk for long-term conditions.

Large language models (LLMs) are advanced artificial intelligence (AI) systems capable of understanding and generating human language [7]. They rely on large-scale training data and predictive learning algorithms to generate contextually coherent responses to user queries [8]. Because they are very easy to use and adaptable, LLMs are increasingly incorporated into medical applications, and their accessibility for both patients and clinicians continues to expand [9]. Despite ongoing ethical concerns surrounding AI-assisted decision-making, emerging evidence shows that LLMs can function as clinically useful decision-support tools under appropriate human supervision, improving both efficiency and quality [10]. We previously evaluated whether LLMs could deliver guideline-compliant recommendations for headache patients [11]. However, their performance in complex, real-world clinical scenarios, particularly those resembling cases presented in the New England Journal of Medicine (NEJM), remains insufficiently characterized. Approximately 38% of headache-related cases remain nonspecific or uncategorized in hospital discharge records, underscoring the diagnostic uncertainty clinicians face in routine practice [12].

To address this gap, the present study systematically evaluated how well contemporary LLMs adhere to NEJM-style clinical guidance when analyzing headache cases. We aimed to determine whether LLMs can meaningfully support clinicians in decision-making, to compare strengths and weaknesses across different model architectures, and to identify areas where knowledge representation or reasoning remains limited. These findings will inform strategies for the safe integration of LLMs into clinical workflows, with the dual goals of enhancing patient safety and reducing clinician workload.

## 2. Methods

### 2.1 Study design

We performed a literature search on The New England Journal of Medicine (NEJM) https://www.nejm.org/, using “headache” and “case record” as keywords, with the timeframe limited to January 2010 to January 2025. Fifteen articles were identified, of which 13 featured headache as one of the top two presenting symptoms. This study quantifies how well LLMs interpret NEJM headache cases and evaluates their effectiveness in assisting with clinical decision-making. We also examined differences in response quality and readability across models to establish a baseline for developing trustworthy AI systems in neurology that may ultimately support more accurate diagnostic outcomes.

This study evaluated the performance of seven LLMs comprising ChatGPT-5, ChatGPT-4o, Deepseek-R1, Gemini-2.5 pro, Grok-3, Hunyuan-T1, and Qwen, in interpreting 13 headache-related clinical cases published in NEJM between 2010 and 2025. These models were selected primarily due to their public accessibility, which increases their practicality for real-world clinical settings [13]. ChatGPT-5 and Qwen require paid subscriptions, while all others offer public access. All data were anonymized and publicly available; no patient-level information was used.

Partial clinical manifestations and laboratory or imaging results were extracted from each case and input into every LLM. The following standardized prompt was used: “Question 1: If you are a doctor, this is a medical case. Please provide the reasoning steps and the three most likely differential diagnoses. Question 2: This is a pathological finding. Please provide the most likely diagnosis. Question 3: What is the treatment for this condition?”.

Two prompting strategies were applied: (1) Ask in sequence (AS): All questions were asked sequentially within one conversation, and the required information was provided step by step. (2) Ask at once (AO): All questions and information were provided simultaneously in a single prompt.

### 2.2 Evaluation process

The 5-point Likert scale is a psychometric tool commonly used to measure attitudes, perceptions, or behaviors [14]. It consists of a series of statements or questions followed by five response options that represent a range of agreement or intensity levels. In this study, it was applied to evaluate six dimensions of each LLM response:

1. Rationality of diagnostic thinking: This evaluated how rational the LLMs are with their diagnostic thinking. (1) Completely irrational (>75% irrational); (2) More irrational than rational; (3) Approximately equal irrational and rational; (4) More rational than irrational (>75% rational); (5) Completely rational.

2. Comprehensiveness of differential diagnosis: This refered to whether one can develop differentials incorporating comorbidities. (1) Completely non-comprehensive; (2) More non-comprehensive than comprehensive (>75% incorrect); (3) Approximately equal non-comprehensive and comprehensive; (4) More comprehensive than non-comprehensive (>75% correct); (5) Completely comprehensive.

3. Diagnostic accuracy: assessing the LLM’s judgment regarding clinical cases correctly. (1) Completely inaccurate; (2) More inaccurate than accurate (>75% inaccurate); (3) Approximately equal inaccurate and accurate; (4) More accurate than inaccurate (>75% accurate); (5) Completely accurate.

4. Completeness of pathological diagnosis: the ability of the LLM to identify and interpret pathological results. (1) Very incomplete (0–25% covered); (2) Incomplete (25–50% covered); (3) Moderately complete (50–75% covered); (4) Complete (>75% covered); (5) Fully complete (100% covered).

5. Clinical management consistency: The agreement between LLMs’ recommendations for treatment options, medications, and treatment guidelines. (1) Completely inconsistent; (2) More inconsistent than consistent (>75% inconsistent); (3) Approximately equal, inconsistent, and consistent; (4) More consistent than inconsistent (>75% consistent); (5) Completely consistent.

6. Supplementary value: Evaluates the comprehensiveness of the knowledge that LLM understands related to the case [15, 16]. (1) No supplementary value (0% added); (2) Low supplementary value (25% added); (3) Moderate supplementary value (50% added); (4) High supplementary value (>75% added); (5) Exceptional supplementary value (100% novel insights). Examples of scores for each dimension were presented in **Supplementary Table 1**.

To assess readability for potential patient comprehension, we applied two widely used metrics: Flesch Reading Ease (FRE) and Flesch-Kincaid Grade Level (FKGL) [17, 18]. Higher FRE scores indicate greater comprehension, while lower FKGL scores indicate suitability for lower-grade reading levels.

The review process was independent and blinded to the specific model identity. To mitigate the “fatigue bias”, the following measures were taken: (1) Reasonably regulated the review time, determined the rest intervals, the number, and duration of reviews. A short break was taken after reviewing the answers of 7 models for one case. (2) Created a comfortable environment by providing a quiet and clean space with appropriate temperature and lighting. (3) Conducted pre-communication: clarified the evaluation criteria and procedures to the judges prior to the evaluation to assist the judges in quickly entering the appropriate state. This procedure was intended to keep their level of attention and their use of the 5-point scale as stable as possible from the first response to the last. Discrepancies (score differences greater than 1 point) were flagged and resolved through a consensus meeting with a fourth senior researcher.

### 2.3 Statistical analysis

Analysis of Variance (ANOVA) with *post hoc* Tukey tests was used to compare model performance. Effect sizes (η^2^) and 95% confidence intervals (CIs) were reported alongside *p*-values. All analyses were conducted using GraphPad Prism 10.1 (GraphPad Software, Boston, MA, USA), with α = 0.05.

## 3. Results

In this study, the performance of seven large-scale language models (ChatGPT-5, ChatGPT-4o, Deepseek-R1, Gemini-2.5 pro, Grok-3, Hunyuan-T1, and Qwen) was evaluated using 13 clinical cases from the NEJM. Each case was assessed under two questioning strategies, and the corresponding model outputs were compared with the original NEJM case content. Evaluation was conducted using a 5-point Likert scale across six dimensions: rationality of diagnostic thinking, comprehensiveness of differential diagnosis, diagnostic accuracy, completeness of pathological diagnosis, clinical management, and supplementary value. Examples of all generated responses were systematically documented in **Supplementary Table 1**.

Inter-rater reliability among the three independent experts was assessed using intraclass correlation coefficients (ICC) calculated in SPSS Statistics 27.0 (IBM Corp, Armonk, NY, USA). A two-way mixed-effects model with a consistency definition was applied, treating subjects as random and raters as fixed, yielding ICC (3,1) for single ratings and ICC (3,3) for the mean of three raters. The ICC for single measurements was 0.893 (95% CI, 0.882–0.903), and the ICC for the average of the three raters was 0.962 (95% CI, 0.957–0.965), indicating good reliability for individual ratings and excellent reliability for the averaged ratings.

### 3.1 Differences between large language models (LLMs)

#### 3.1.1 Diagnostic accuracy differed significantly across models under the same questioning strategy

For the AS (ask in sequence) group, ChatGPT-4o demonstrated significantly higher diagnostic accuracy than Grok-3 (*p* = 0.0488) (Fig. 1A). No other pairwise comparisons reached statistical significance, and a similar absence of significant differences was observed among models in the AO (Fig. 1B).

**Fig. 1.** Comparison of diagnostic accuracy among different models in the AS and AO groups. (A) ChatGPT-4o had the highest accuracy score, and Grok-3 had the lowest accuracy score. The average scores of other groups were greater than 3 points. (B) Qwen had the lowest accuracy score. However, there was no significant difference compared with other groups. Asterisks (*) indicate statistical significance at *p * < 0.05. AS represents ask in sequence group; AO represents ask at once group.

#### 3.1.2 Supplementary value differed significantly across models under the same questioning strategy

For the AS group, Grok-3 demonstrated a significantly higher supplementary value score than ChatGPT-5 (*p* = 0.0009) and Hunyuan-T1 (*p* = 0.0294) (Fig. 2A).

For the AO group, ChatGPT-5 scored significantly lower than Deepseek-R1 (*p* = 0.0035) (Fig. 2B).

**Fig. 2.** Comparison of supplementary values among different models in the AS and AO groups. (A) Grok-3 had the highest supplement value, while ChatGPT-5 had the lowest supplement value. (B) Deepseek-R1 had the highest supplement value, and ChatGPT-5 had the lowest supplement value. Asterisks (*, **, and ***) indicate statistical significance at *p * < 0.05, *p * < 0.01, and *p * < 0.001, respectively. AS represents the ask in sequence group; AO represents ask at once group.

All other pairwise comparisons within both questioning strategies did not reach statistical significance (adjusted* p * > 0.05 for all other model-strategy combinations) with respect to supplementary values.

One-way ANOVA showed no significant differences among the seven models in either questioning strategy for the rationality of diagnostic thinking (AS group *p* = 0.2248, AO group *p* = 0.4394), comprehensiveness of differential diagnosis (AS group *p* = 0.3930, AO group *p* = 0.7104), completeness of pathological diagnosis (AS group *p* = 0.9258, AO group *p* = 0.9903), and clinical management (AS group *p* = 0.7378, AO group *p* = 0.8518) (Figs. 3A–D,4A–D). The complete visual comparison and summary of all evaluation outcomes across all models is provided in **Supplementary Fig. 1**.

**Fig. 3.** Comparison among various models in the AS group. (A) Comparing the rationality scores of diagnostic ideas among various models, Hunyuan-T1 and Deepseek-R1 performed worse than other models, but the differences were not significant. (B) Comparison of the comprehensiveness of differential diagnosis scores among the models shows that all models performed poorly. (C) Comparison of pathological diagnosis completeness scores among various models showed that each model performed well. (D) Comparison of clinical management consistency scores among various models showed that Hunyuan-T1 performed the worst. AS denotes ask in sequence group.

**Fig. 4.** Comparison among various models in the AO group. (A) Comparing the rationality scores of diagnostic ideas among various models, Hunyuan-T1 performed worse than other models. (B) Comparison of the comprehensiveness of differential diagnosis scores among the models shows that all models performed poorly. (C) Comparison of pathological diagnosis completeness scores among various models showed that each model performed well. (D) Comparison of clinical management consistency scores among various models showed that ChatGPT-4o performed the worst. AO represents ask at once group.

### 3.2 Differences between questioning strategies

In terms of diagnostic accuracy, only the Grok-3 model showed a significant difference between strategies, with the AO group scoring higher than the AS group (*p* = 0.0273). For supplementary value, several models performed significantly better under the AS strategy. Specifically, the AS group scored higher than the AO group for ChatGPT-5 (*p* = 0.0007), ChatGPT-4o (*p* = 0.0102), Gemini-2.5 Pro (*p* = 0.0020), Grok-3 (*p* = 0.0004), and Qwen (*p* = 0.0087). All the remaining comparisons did not reach statistical significance (Fig. 5).

**Fig. 5.** Comparison of scoring outcomes across models under two questioning strategies. (A) ChatGPT-5: The AS group achieved higher supplementary value, whereas the AO group demonstrated higher diagnostic accuracy. (B) ChatGPT-4o: The AS group outperformed the AO group in most evaluation dimensions. (C) DeepSeek-R1: The AS group showed higher supplementary value, while the AO group performed better across the remaining dimensions. (D) Gemini-2.5 Pro: The AS group exhibited higher supplementary value, with similar performance between strategies in other dimensions. (E) Grok-3: The AS group had higher supplementary value, whereas the AO group demonstrated significantly higher diagnostic accuracy. (F) The AS group scored higher in supplementary value and diagnostic thinking, while the AO group showed slightly better performance in other dimensions. (G) Qwen: The AS group consistently outperformed the AO group, particularly in supplementary value. Asterisks (*, **, and ***) indicate statistical significance at *p *< 0.05, *p *< 0.01, and *p * < 0.001, respectively. AS represents ask in sequence group; AO represents ask at once group.

### 3.3 Differences in LLM’s performance across different cases

The scores across all the evaluation dimensions for 13 NEJM cases were summarized in **Supplementary Fig. 2**. LLMs performance was markedly lower for Cases C8 and C11, which consistently received the lowest scores among all cases. In contrast, higher performance was observed for Cases C2, C10, and C13. Detailed statistical outputs for all case-level comparisons were provided in **Supplementary Figs. 3,4,5,6,7,8**.

## 4. Differences in FRE

### 4.1 Impact of the seven models on FRE within the same questioning strategy

In the AS group, the Gemini 2.5 pro achieved significantly higher FRE scores than the ChatGPT-4o (*p * < 0.0001), Deepseek-R1 (*p* = 0.0014), Grok-3 (*p* = 0.0218), and Hunyuan-T1 (*p * < 0.0001). Qwen also showed a higher FRE score than ChatGPT-4o (*p* = 0.0285) and Hunyuan-T1 (*p* = 0.0347) (Fig. 6A).

In the AO group, both Deepseek-R1 (*p* = 0.0340) and Hunyuan-T1 (*p* = 0.0022) scored significantly lower than that of the Gemini 2.5 pro. Additionally, the Hunyuan-T1 scored lower than the Qwen (*p* = 0.0369). None other pairwise comparisons reached statistical significance (Fig. 6B).

**Fig. 6.** Comparison of FRE among different models in the AS and AO groups. (A) The FRE value of Gemini-2.5 pro was the highest and significantly higher than that of other groups, while the FRE value of ChatGPT-4o was the lowest. (B) The FRE value of Gemini-2.5 pro was the highest, and the FRE value of Hunyuan-T1 was the lowest. Asterisks (*, ** and ****) indicate statsistical significance at *p *< 0.05, *p * < 0.01, and *p * < 0.0001, respectively. AS: ask in sequence; AO: ask at once; FRE: Flesch Reading Ease.

### 4.2 Different questioning strategies have an impact on FRE

In Gemini-2.5 pro, the AS group demonstrated a significantly higher FRE than the AO group (*p *= 0.0166). No significant differences were observed between questioning strategies for any of the other models (Fig. 7A–G).

**Fig. 7.** Comparison of the FRE value across models under two questioning strategies. (A) ChatGPT-5: Comparison of the FRE value between the AS and AO groups. (B) ChatGPT-4o: Comparison of the FRE value between the AS and AO groups. (C) DeepSeek-R1: Comparison of the FRE value between the AS and AO groups. (D) Gemini-2.5 Pro: Comparison of the FRE value between the AS and AO groups. (E) Grok-3: Comparison of the FRE value between the AS and AO groups. (F) Hunyuan-T1: Comparison of the FRE value between the AS and AO groups. (G) Qwen: Comparison of the FRE value between the AS and AO groups. For each model, FRE values were compared between the ask-in-sequence (AS) and ask-at-once (AO) strategies. Bars represent mean FRE across cases, and error bars indicate the 95% confidence interval (95% CI). In ChatGPT-4o, the AO group showed a higher FRE value than the AS group. For all other models, the AS group produced higher FRE values, with a significant difference observed only for Gemini-2.5 pro. Asterisks (*) indicate statistical significance at *p * < 0.05. AS: ask in sequence; AO: ask at once.

## 5. Differences in FKGL

### 5.1 Impact of the seven models on FKGL within the same questioning strategy

In the AS group, ChatGPT-4o produced significantly higher FKGL score compared with Deepseek-R1 (*p* = 0.0004), Gemini 2.5 pro (*p * < 0.0001), Hunyuan-T1 (*p* = 0.0369), and Qwen (*p* = 0.0032). No other pairwise comparisons reached statistical significance, and no significant differences were observed among models in the AO group (Fig. 8A,B).

**Fig. 8.** Comparison of FKGL scores among different models in the AS and AO groups. (A) The FKGL value of ChatGPT-4o was the highest and significantly higher than that of other groups, while the FKGL value of Gemini-2.5 pro was the lowest. (B) There was no significant difference in FKGL values among the AO groups. Asterisks (*, **, *** and ****) indicate statistical significance at *p * < 0.05, *p * < 0.01, *p * < 0.001 and *p *< 0.0001, respectively. AS: ask in sequence; AO: ask at once; FKGL: Flesch-Kincaid Grade Level.

### 5.2 Different questioning strategies have an impact on FKGL

For ChatGPT-4o, the AS group demonstrated a significantly higher FKGL than the AO group (*p *= 0.0012). In contrast, for the Gemini-2.5 pro, the AS group showed a significantly lower FKGL than the AO group (*p* = 0.0022). No other significant differences were observed across models (Fig. 9A–G).

**Fig. 9.** Comparison of the FKGL of different models under two questioning strategies. (A) ChatGPT-5: Comparison of the FKGL between the AS and AO groups. (B) ChatGPT-4o: Comparison of the FKGL between the AS and AO groups. (C) DeepSeek-R1: Comparison of the FKGL between the AS and AO groups. (D) Gemini-2.5 Pro: Comparison of the FKGL between the AS and AO groups. (E) Grok-3: Comparison of the FKGL between the AS and AO groups. (F) Hunyuan-T1: Comparison of the FKGL between the AS and AO groups. (G) Qwen: Comparison of the FKGL between the AS and AO groups. For each model, FKGL values are compared between the ask-in-sequence (AS) and ask-at-once (AO) strategies. Bars represent mean FKGL across cases, and error bars indicate the 95% confidence interval (95% CI). For ChatGPT-4o, the FKGL value of the AS group was significantly higher than that of the AO group. For other models, the FKGL values of the AO group were all higher than those of the AS group. Asterisks (**) indicate statistical significance at *p * < 0.01. AS: ask in sequence; AO: ask at once; FKGL: Flesch-Kincaid Grade Level.

## 6. Discussion

This study provides the first comprehensive evaluation of seven state-of-the-art LLMs in analyzing headache-related clinical cases. Our results reveal the capabilities and limitations of these models in supporting neurological decision-making and highlight key directions for future development.

For clinical practice, the performance of LLMs was most different in three areas. First, in diagnostic accuracy, ChatGPT-4o outperformed Grok-3 within the AS group, and generally achieved higher scores across several other evaluation dimensions. This advantage may reflect access to higher-quality and more medically relevant training data rather than differences in model size [19]. Second, in supplementary value, both Grok-3 and Deepseek-R1 showed particularly strong performance. Grok-3’s advantage may stem from its real-time Web access, which can provide up-to-date contextual information, while Deepseek-R1 performed best under AO prompting, suggesting strong integration of complex multimodal clinical information [20]. Third, in readability, Gemini-2.5 Pro consistently achieved the highest FRE and lowest FKGL (except in AO-FKGL), indicating that its outputs were the easiest to read, a meaningful advantage for clinician-patient communication and clinical documentation.

From all models, regardless of AS or AO prompting, there was no significant difference in the completeness of differential diagnosis, completeness consistency of pathology diagnosis, and clinical management. Compared with human experts, all LLMs performed well in terms of the rationality of their diagnostic thinking processes, clinical management, and the completeness of pathological diagnoses; this indicates that LLMs can be of some help to clinicians when managing diseases [21].

However, a major and consistent limitation across all models was their unsatisfactory performance in the comprehensiveness of differential diagnoses. The original text’s differential diagnosis primarily focuses on the patient’s systemic symptoms, placing greater emphasis on clinical manifestations (such as fever, headache, and pregnancy), while also including life circumstances such as homelessness. On the contrary, LLMs analyze the patient’s most significant symptoms and focus on their causes more. This points to a basic lack in its clinical thinking capacity for headache ailments that call for a fine blending of overlapping signs and risk factors. The models’ constantly poor results when there are psychiatric comorbidities or “red flag” symptoms (C8, C11) are very concerning, as they show a potentially dangerous “red flag blindness”. This inclination for going back to commonly found primary headache patterns in spite of having record evidence about risk factors related to secondary headaches presents an immediate threat to patients, so humans have to keep an eye on it [22].

Our findings regarding prompting strategies offer additional insight. AS prompting, which mimics stepwise clinical reasoning, generally increased supplementary value and readability, aligning with the need for transparent and traceable thought processes in clinical workflows. However, increased verbosity did not guarantee improved accuracy; for instance, Grok-3 produced more detailed but clinically incorrect reasoning under AS, illustrating a risk of coherent yet incorrect “hallucinations” expressed with unwarranted confidence [23]. Meanwhile, the questioning strategy can also affect the difficulty of reading. Although the FKGL of ChatGPT-4o was significantly higher in the AS group, the FKGL of the other models was higher in the AO group, and the corresponding FRE values were also the same. This indicates that the one-time questioning strategy may unintentionally increase the complexity of grammar, which requires the interface to be optimized to adapt to clinical applications. At the same time, we found that whether it was the AO group or the AS group, the differences in several other indicators were very small, which indicates that the questioning strategy has a limited impact on clinical management.

Another important observation is the potential for embedded biases in LLM outputs, which may manifest as models inadvertently associating certain illnesses with specific demographic or social groups, thereby leading to unfair or unsafe recommendations [24]. Our results highlight a significant problem in the current LLMs—they cannot differentiate between benign primary headaches and more complicated or rare secondary headaches, which is a concerning shortfall when it comes to incorrectly identifying a secondary headache that can harm patient safety. We need to do domain-specific fine-tuning with datasets containing uncommon and strange presentations of headache, since our present training data has a statistical bias for usual types of headaches. From the limitation, we can see the poor performance of the model on cases having psychiatric comorbidities and red flags. Clinicians usually use SNNOOP10 red flags to determine if it’s at risk [25]. Our findings directly address three major challenges facing LLMs: data scarcity [26], situational nuances [27, 28], and evidence integration [29]. These findings emphasize that LLMs should be used as decision support tools rather than stand-alone diagnostic tools, especially in high-risk situations. At the same time, LLMs still exhibit variability in performance on neurological knowledge tasks, highlighting the need for careful, specialty-specific validation [30].

As with any major tech advancement, the addition of LLMs into clinical workflows demands a forward-thinking attitude on ethical and safety issues [31]. Patients’ private information is the most important thing. Outsourcing confidential data to closed-source application programming interfaces poses a great threat of misuse or unintended use for training models, which indicates hospitals can also choose to host their own open-source system instead. Moreover, the legal liability for all sorts of negative results resulting from making choices by means of an LLM remains uncertain; therefore, it should be emphasized repeatedly that these are merely aids used when deciding on medical care decisions, they cannot independently make such decisions themselves and bear no responsibility over any outcome. The pretraining has bias which would lead to biases after being used clinically. Therefore, mitigation measures were firstly performed, fine-tuning with selected relevant domain datasets and then continuous monitoring is carried out. Regarding the question of data security issues using closed source, it highlights why hospitals could consider maintaining in-house open-source alternatives so that all rules for keeping patients’ information safe get followed properly [32]. Therefore, to resolve these problems, it would take more than just technical fixes like making models clearer and making sure the data is fair. We also need to have strong ethical guidelines with clear standards for informed consent and responsibility and get everyone in the industry to agree on adding mandatory ethical reviews when we put these models into use in clinical settings [33].

## 7. Conclusions

In summary, although several LLMs show promise in selected aspects of headache case analysis, they remain insufficient for standalone clinical use, particularly in detecting complex or atypical secondary headaches where diagnostic precision is critical. LLMs should currently serve as supportive tools rather than replacements for clinicians. Closing the performance and safety gap will require collaboration between neurologists and AI developers, along with specialized training datasets, especially those enriched for rare or high-risk headache presentations. Future systems must incorporate validated clinical reasoning modules, safety mechanisms, and comprehensive ethical safeguards throughout the model life cycle. Ultimately, the transformative potential of LLMs in reducing the global burden of headache disorders can only be realized if they are deployed as rigorously validated, human-supervised tools within frameworks that prioritize patient safety above all else.

## Supplementary Material

Supplementary material associated with this article can be found, in the online version, at https://files.jofph.com/ files/article/2031991005798907904/attachment/ Supplementary%20material.docx.


