# Supplementary material for: Benchmark evaluation of large language models for clinical decision support in headache management
Source: J Oral Facial Pain Headache. 2026 Mar 12;40(2):140–50. doi: 10.22514/jofph.2026.029 (PMC13036623; doi:10.22514/jofph.2026.029)
Supplement: Supplementary file 1 [file Supplementary-material.docx]

Supplementary material


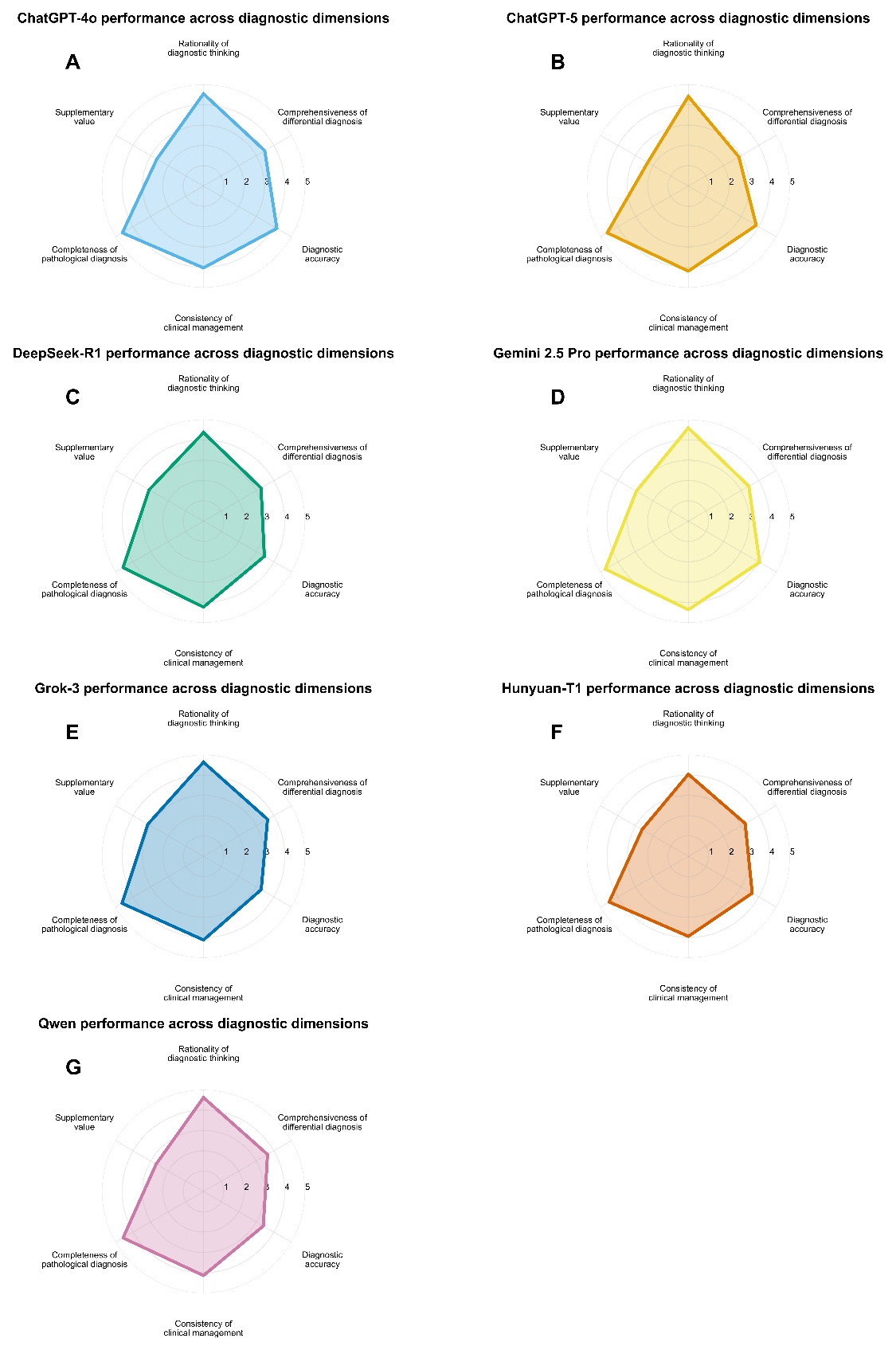


**Supplementary Fig. 1. Score notes of different large language models (LLMs) in six different aspects.** Radar plots of diagnostic performance across diagnostic dimensions for seven large language models. (A–G) show ChatGPT-4o, ChatGPT-5, DeepSeek-R1, Gemini 2.5 Pro, Grok-3, Hunyuan-T1 and Qwen, respectively. For each model, expert raters scored the generated answers on a 5-point Likert scale across six dimensions: rationality of diagnostic thinking, comprehensiveness of differential diagnosis, diagnostic accuracy, consistency of clinical management, completeness of pathological diagnosis, and supplementary value. Larger shaded areas indicate better overall diagnostic performance.


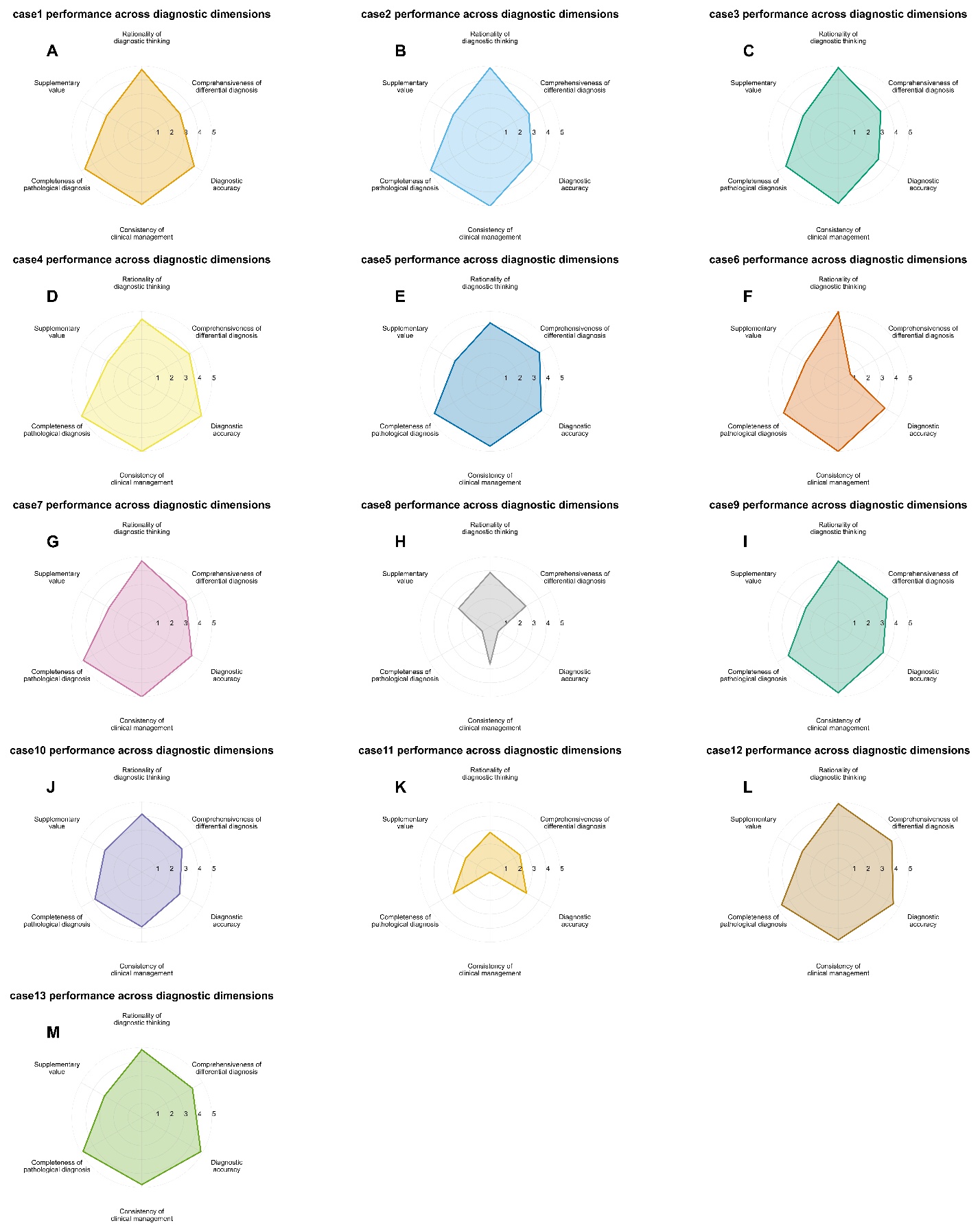


**Supplementary Fig. 2. Score notes of different Cases in six different aspects.** (A–M) show Case 1–13, respectively: Case 1, Plasmodium falciparum malaria; Case 2, recurrent meningococcal meningitis; Case 3, Susac’s syndrome; Case 4, neurocysticercosis; Case 5, multiple intracranial aneurysms; Case 6, parainfectious meningoencephalomyeloradiculitis; Case 7, Strongyloides stercoralis hyperinfection syndrome; Case 8, Neuro-IRIS; Case 9, acute HIV infection; Case 10, parasitic infection; Case 11, schizophrenia; Case 12, primary CNS lymphoma; and Case 13, small-cell variant of glioblastoma. For each case, generated answers were scored on a 5-point Likert scal across six dimensions: rationality of diagnostic thinking, comprehensiveness of differential diagnosis, diagnostic accuracy, consistency of clinical management, completeness of pathological diagnosis, and supplementary value. Larger shaded areas indicate better overall performance in that case.


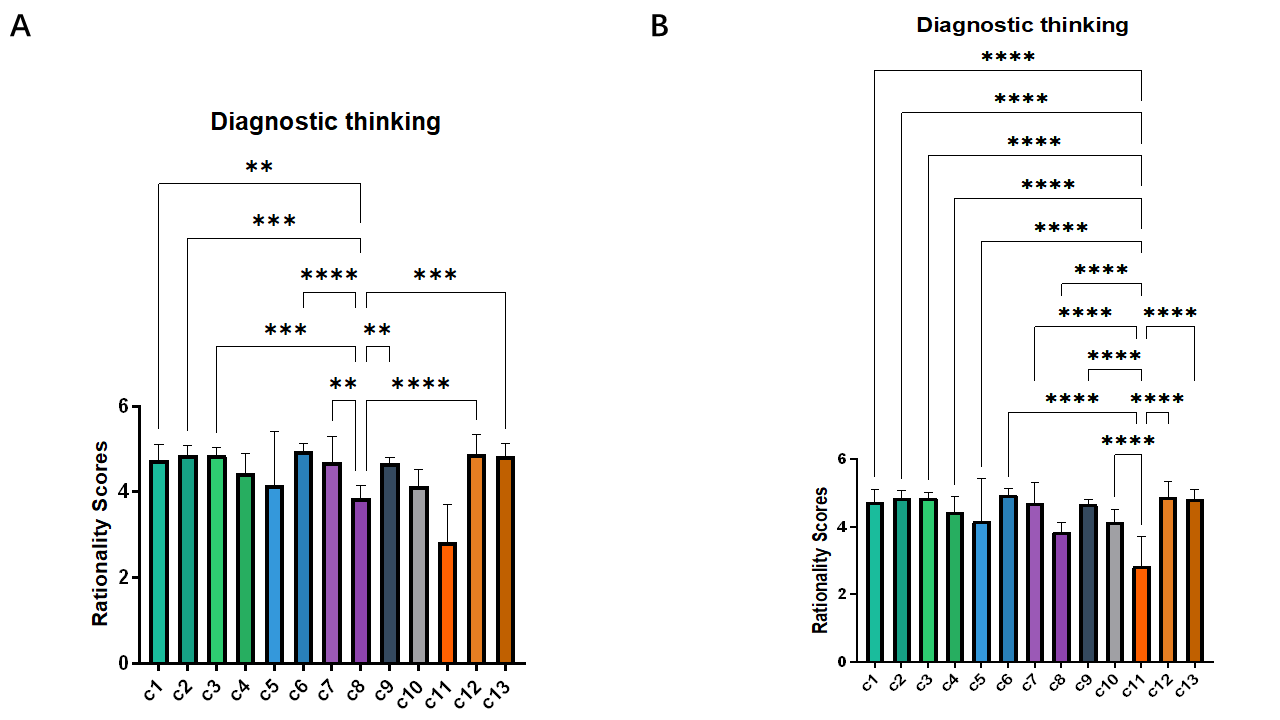


**Supplementary Fig. 3. Case wise rationality scores for diagnostic thinking.** (A) Case C8 shows consistently low comprehensiveness scores and differs significantly from multiple other cases in post hoc comparisons. (B) Case C11 shows consistently low comprehensiveness scores and differs significantly from multiple other cases in post hoc comparisons. Mean scores for the rationality of diagnostic thinking across the 13 diagnostic cases (c1–c13). Bars show the average Likert score (1 to 5) for each case, and error bars indicate variability between expert raters. Asterisks above the bars represent statistically significant pairwise differences between cases from *post hoc* multiple comparisons after one way analysis of variance (more asterisks indicate a smaller *p* value).


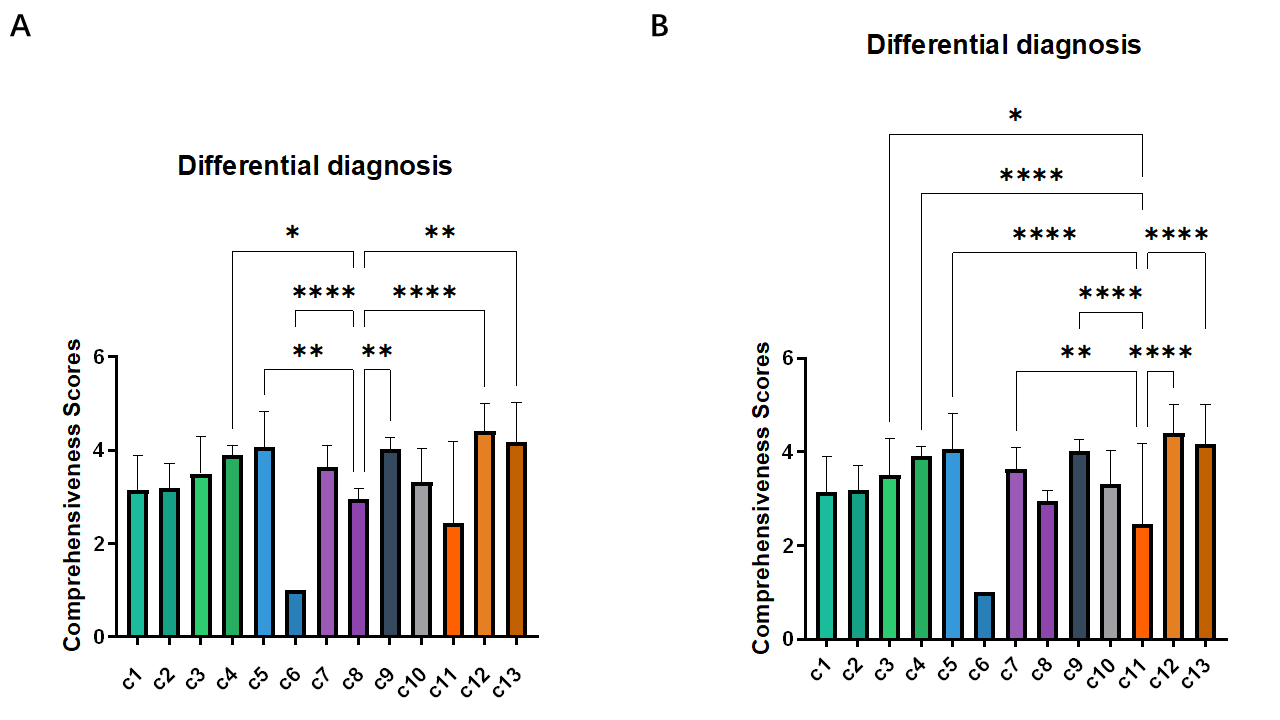


**Supplementary Fig. 4. Case level scores for the comprehensiveness of differential diagnosis.** (A) Case C8 shows consistently low comprehensiveness scores and differs significantly from multiple other cases in post hoc comparisons. (B) Case C11 shows consistently low comprehensiveness scores and differs significantly from multiple other cases in post hoc comparisons. Bars show the mean scores for the “comprehensiveness of differential diagnosis” dimension for each of the 13 cases (C1–C13), using a 5-point Likert scale where higher values indicate a more complete and structured differential diagnosis. Error bars represent variation between expert raters. Where present, asterisks above the bars indicate statistically significant differences between cases based on one way analysis of variance with *post hoc* multiple comparisons.


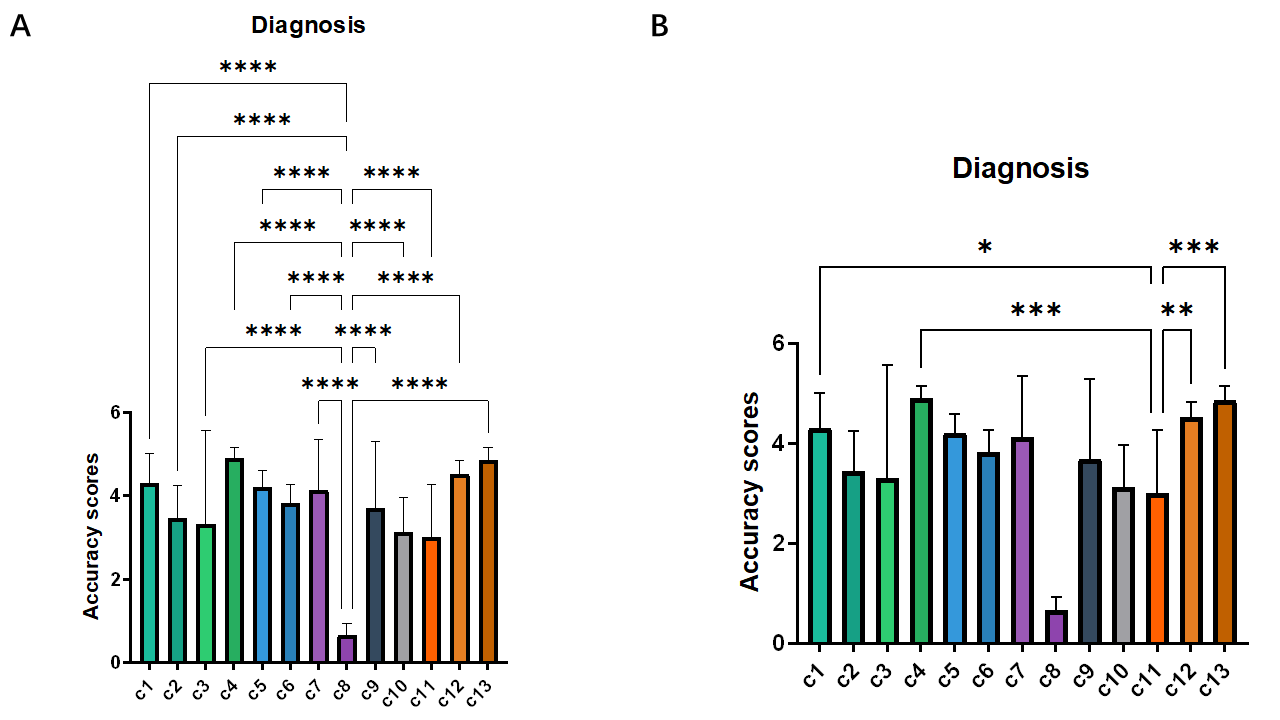


**Supplementary Fig. 5. Case-level scores for diagnostic accuracy.** (A) Case C8 shows consistently low comprehensiveness scores and differs significantly from multiple other cases in post hoc comparisons. (B) Case C11 shows consistently low comprehensiveness scores and differs significantly from multiple other cases in post hoc comparisons. Mean scores for the “diagnostic accuracy” dimension for each of the 13 cases (C1–C13), rated on a 5-point Likert scale where higher values indicate closer agreement with the reference diagnosis. Error bars represent variability between expert raters. Asterisks above the brackets indicate statistically significant differences between cases based on one-way analysis of variance followed by *post hoc* multiple comparisons (more asterisks indicate a smaller *p* value).


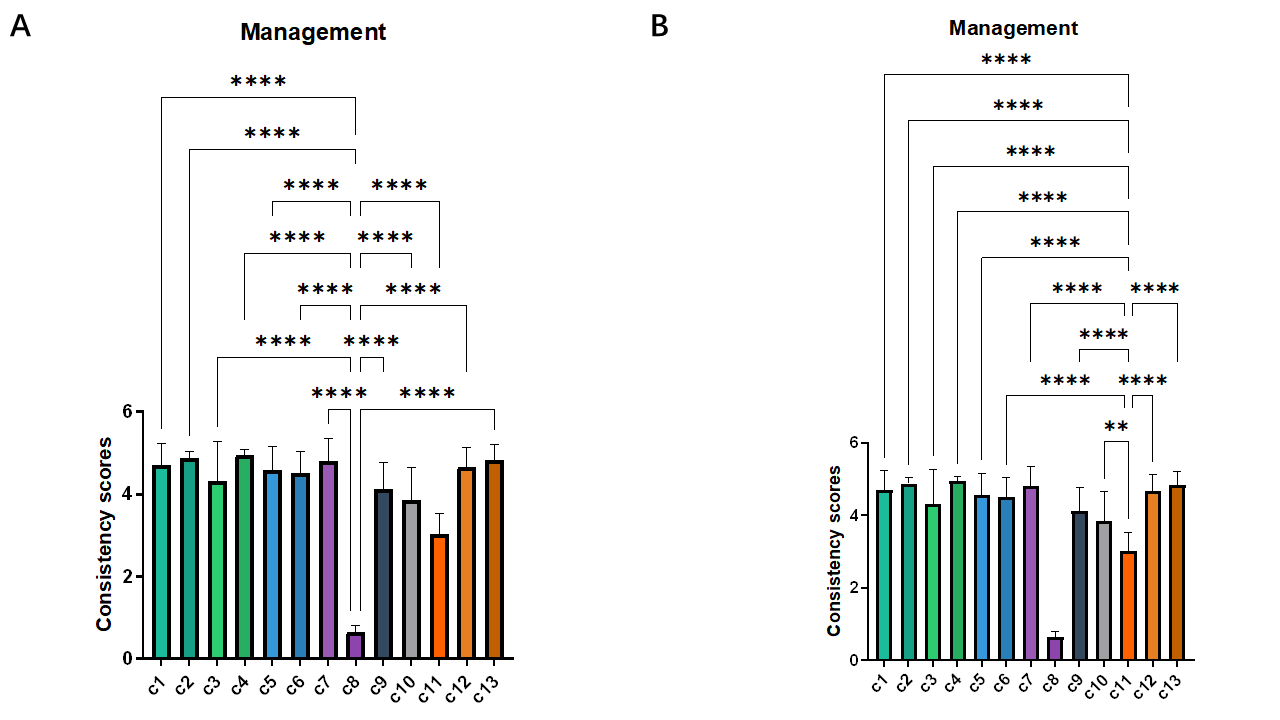


**Supplementary Fig. 6. Case level scores for consistency of clinical management.** (A) Case C8 shows consistently low comprehensiveness scores and differs significantly from multiple other cases in post hoc comparisons. (B) Case C11 shows consistently low comprehensiveness scores and differs significantly from multiple other cases in post hoc comparisons. Mean scores for the “consistency of clinical management” dimension for each of the 13 cases (C1–C13), rated on a 5-point Likert scale where higher values indicate closer agreement with guideline-based management. Error bars represent variability between expert raters. Asterisks above the brackets indicate statistically significant differences between cases based on one way analysis of variance followed by *post hoc* multiple comparisons (more asterisks indicate a smaller *p* value).


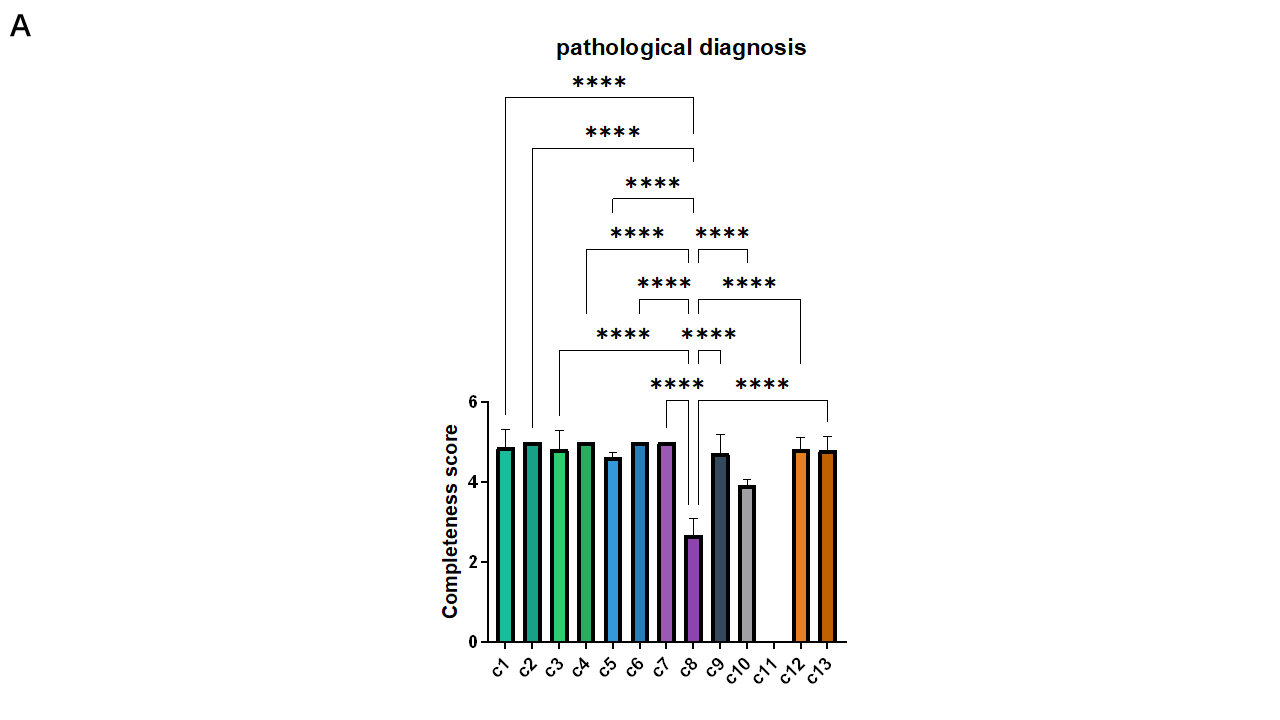


**Supplementary Fig. 7. Case level scores for completeness of pathological diagnosis.** Mean scores for the “completeness of pathological diagnosis” dimension for each of the 13 cases (C1–C13), rated on a 5-point Likert scale where higher values indicate a more complete and clinically adequate pathological interpretation. Bars show the mean completeness score and error bars represent variability between expert raters. Asterisks above the brackets indicate statistically significant differences between cases based on one way analysis of variance followed by *post hoc* multiple comparisons (more asterisks indicate a smaller *p* value).


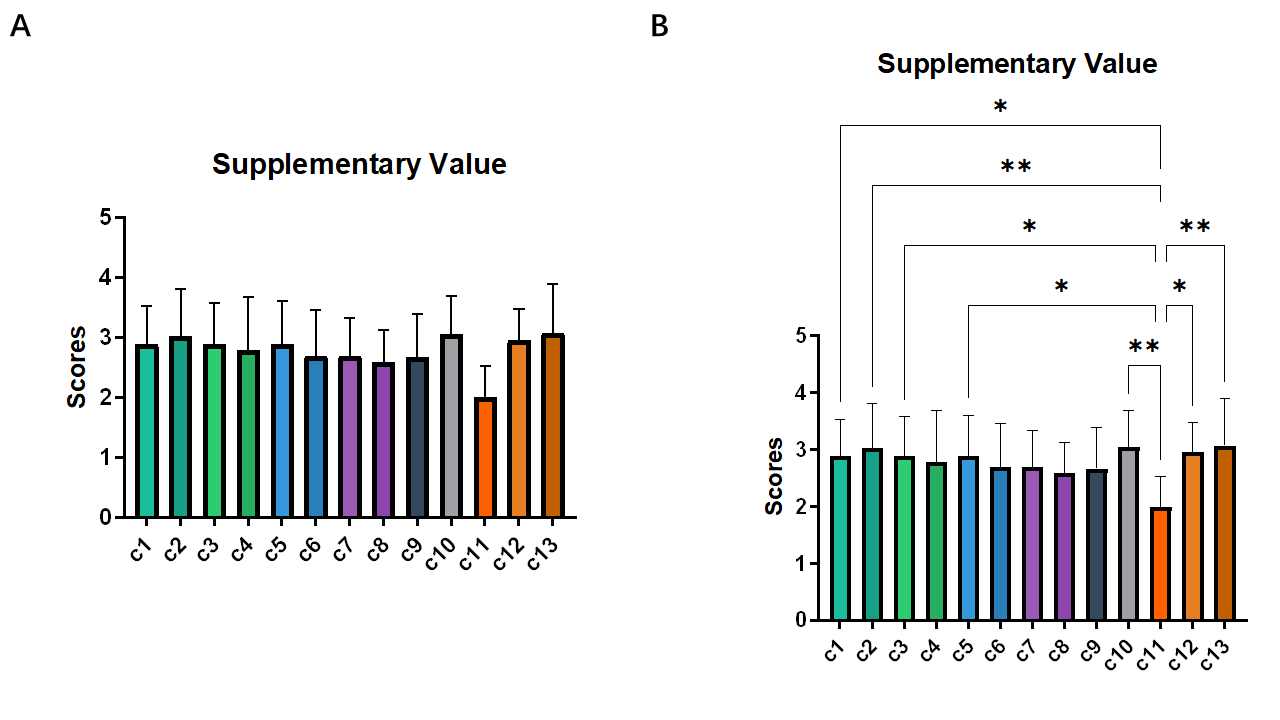


**Supplementary Fig. 8. Case level scores for supplementary value.** (A) Case C8 did not differ significantly from the other cases in supplementary value. (B) Case C11 shows consistently low comprehensiveness scores and differs significantly from multiple other cases in post hoc comparisons. Mean scores for the “supplementary value” dimension for each of the 13 cases (C1–C13), rated on a 5 point Likert scale where higher values indicate greater added clinical or educational value beyond the original case description. Bars represent the mean score for each case and error bars show variability between expert raters. Asterisks above the brackets indicate statistically significant differences between cases based on one way analysis of variance followed by *post hoc* multiple comparisons (more asterisks indicate a smaller *p* value).

Supplementary Table 1. Examples of scores for each dimension.

| Dimension | New England Journal of Medicine original article | The response of LLM | Score |
| --- | --- | --- | --- |
| Rationality of diagnostic thinking | This previously healthy 28-year-old woman presented with severe headache, nausea, vomiting, myalgias, and skin findings. The presence of an evolving petechial rash, with new lesions developing over a period of only a few hours, suggests a rapidly progressive disease requiring prompt recognition and management. A timeline of this patient’s presentation highlights the key features of this case (Fig. 2); these features were identified solely through history taking and physical examination, underscoring the crucial role of basic bedside clinical skills in the early detection of potential medical emergencies. In this patient, there appears to be a confluence of three disease processes causing a systemic illness with manifestations involving the skin and central nervous system. At the time of presentation to the emergency department, the patient’s heart rate was higher than 90 beats per minute and her white-cell count was greater than 12,000 per cubic millimeter. With the presence of these two findings, she met the criteria for the systemic inflammatory response syndrome (SIRS). Although thrombocytopenia was present, there was no evidence of overt disseminated intravascular coagulation; the d-dimer level was only mildly elevated and the fibrinogen level, prothrombin time, and activated partial-thromboplastin time were normal. Hypokalemia, hypomagnesemia, hypophosphatemia, and hyponatremia and the presence of albumin, red cells, and renal tubular cells in the urine of this previously healthy patient also raise the possibility of sepsis. | • Reasoning steps:  Symptoms: The 28-year-old woman presented with acute onset of fever, severe headache, myalgias, petechial rash, mild neck stiffness, and rapid clinical progression.  • Examination: Petechiae and purpura are strongly suggestive of meningococcemia.  • Laboratory: Blood culture Gram stain shows Gram-negative, coffee-bean shaped diplococci, both intra- and extracellular. This is highly characteristic of Neisseria meningitidis.  Predisposing factor: Complement deficiency (C8 deficiency) predisposes to recurrent Neisseria infections. Key findings leading to diagnosis:  • Acute febrile illness with meningismus  • Petechial and purpuric rash (suggestive of meningococcemia)  • Gram stain: Gram-negative diplococci inside neutrophils  • Blood culture positivity | LLM reasoning in the order of “symptoms → signs → laboratory → predisposing factors”: First, we summarized the acute onset of fever, severe headache, myalgia, rash and mild neck stiff, then emphasized that petechiae and ecchymosis suggested neisseria meningitidis bacteremia, and then we considered the gram-negative diplococcus nephriformis in blood culture as a highly specific clue. Finally, we considered C8 deficiency as a key factor to explain recurrent infection and susceptibility, so as to reasonably summarize “invasive neisseria meningitidis infection”. |
| Comprehensiveness of differential diagnosis | This previously healthy 28-year-old woman presented with severe headache, nausea, vomiting, myalgias, and skin findings. The presence of an evolving petechial rash, with new lesions developing over a period of only a few hours, suggests a rapidly progressive disease requiring prompt recognition and management. A timeline of this patient’s presentation highlights the key features of this case (Fig. 2); these features were identified solely through history taking and physical examination, underscoring the crucial role of basic bedside clinical skills in the early detection of potential medical emergencies. In this patient, there appears to be a confluence of three disease processes causing a systemic illness with manifestations involving the skin and central nervous system. At the time of presentation to the emergency department, the patient’s heart rate was higher than 90 beats per minute and her white-cell count was greater than 12,000 per cubic millimeter. With the presence of these two findings, she met the criteria for the systemic inflammatory response syndrome (SIRS). Although thrombocytopenia was present, there was no evidence of overt disseminated intravascular coagulation; the d-dimer level was only mildly elevated and the fibrinogen level, prothrombin time, and activated partial-thromboplastin time were normal. Hypokalemia, hypomagnesemia, hypophosphatemia, and hyponatremia and the presence of albumin, red cells, and renal tubular cells in the urine of this previously healthy patient also raise the possibility of sepsis.  Vasculopathy  The second readily evident disease process in this patient revolves around her skin findings of petechiae and a purpuric lesion; these findings are suggestive of a vasculopathy. It is perhaps equally important to note the absence of bullae, gangrenous changes, and crepitance, findings that would suggest tissue necrosis or infarction, and the absence of generalized purpura, a finding that would be indicative of purpura fulminans. Vasculopathy in the presence of sepsis, or septic vasculopathy, often results in purpuric lesions and may be caused by a variety of organisms, including *N. meningitidis,* which accounts for approximately half the cases among nonimmunosuppressed patients. Septic vasculopathy may occur within 24 hours after the onset of illness, as was observed in this patient, and biopsy of a lesion characteristically reveals intravascular fibrin thrombi of the superficial and deep-skin vessels, some with fibrinoid necrosis. These findings may be associated with bacterial endotoxemia.  Meningitis  In addition to SIRS and vasculopathy, this patient had acute meningitis, as indicated by the abrupt onset of severe headache and the neutrophilic pleocytosis in the cerebrospinal fluid (CSF). The following clinical findings that are often associated with bacterial meningitis were noticeably absent in this case: mental-status changes, a CSF neutrophil count of greater than 1000 cells per cubic millimeter, visible organisms on Gram’s staining, hypoglycorrhachia (a low CSF glucose level), and an elevated CSF protein level. However, it is important to recognize that one or more clinical or laboratory signs or symptoms that are typical of meningitis—such as fever, nuchal rigidity, altered mental status, and a CSF white-cell count of greater than 1000 per cubic millimeter—may be absent in a substantial number of patients with this diagnosis, and the CSF glucose and protein levels may be normal. Similarly, of adults with meningococcal meningitis, impaired consciousness may be absent in 49% and Gram’s staining of CSF may be negative for organisms in 11%. The relatively mild CSF abnormalities in this patient may also indicate that she presented very early in the course of bacterial meningitis. Given the broad range of clinical and laboratory presentations of patients with bacterial meningitis and the likelihood of septic vasculopathy in this patient, a bacterial cause of meningitis remains the most likely diagnosis.  *Bacterial Causes of Meningitis*  *Streptococcus pneumoniae* should be a serious consideration in this patient because it is the most common cause of bacterial meningitis in adults, although it is not likely to be associated with vasculopathy. *Haemophilus influenzae* is a very uncommon cause of bacterial meningitis and vasculopathy in adults. *Staphylococcus aureus* can be associated with bacteremia and vasculopathy, but community-acquired meningitis and purpura are rarely caused by this organism. *N. gonorrhoeae* bacteremia can be associated with vasculopathy, but it would be unusual for this organism to cause meningitis; also, given the reported lack of sexual activity in this patient, this diagnosis is unlikely. *Capnocytophaga canimorsus* is a gramnegative bacillus associated with exposure to dogs. *C. canimorsus* infection may be manifested by sepsis, vasculopathy, and meningitis but often occurs in the presence of asplenia and dog bite; neither of these features was an apparent risk factor in this patient. Although *Streptococcus pyogenes* has been associated with meningitis and vasculopathy, group A streptococcal meningitis usually occurs in the presence of otitis media or sinusitis. *Rickettsia rickettsii*, the agent of Rocky Mountain spotted fever, can cause severe vasculopathy and occasionally meningitis; however, a rash developed within the first 24 hours after illness onset and the patient had no reported exposure to ticks, and thus an infection caused by this organism seems unlikely. Anthrax should be in the differential diagnosis of any illness with an abrupt onset that is characterized by sepsis and vasculopathy, but the absence of a fulminant disease course associated with shock and hemorrhagic meningitis makes this diagnosis very unlikely. Bacteremia due to *Kingella kingae*, a gram-negative bacillus, can be associated with signs and symptoms similar to those seen in this patient; however, *K. kingae* infection occurs primarily in children and is rarely complicated by meningitis. Other gram-negative bacilli, such as *Pseudomonas aeruginosa,* are also in the differential diagnosis; it is rare for *P. aeruginosa* to cause community-acquired meningitis, and when it does, it usually affects patients with underlying chronic medical conditions.  *N. meningitidis* is associated with a rapidly progressive, sometimes fulminant illness characterized by sepsis, petechial rash, purpura, and meningitis. This organism is the second most common cause of bacterial meningitis in adults, and most cases occur sporadically. Although the incidence of *N. meningitidis* infection is highest among children and young adults, the burden of disease is heaviest among adults 25 through 64 years of age; this patient is 28 years of age. Of adults with bacterial meningitis and concurrent rash, two thirds receive a diagnosis of meningococcal disease and most have a petechial rash. Of adults with meningococcal meningitis, 49% seek medical attention less than 24 hours after the onset of symptoms and 64% have a rash, of whom the majority have petechiae and one fourth have purpura, ecchymoses, or both15; these features are consistent with this patient’s clinical presentation. Although this patient had received vaccination against *N. meningitidis* serogroups A, C, Y, and W-135, it is possible that she was infected with another serogroup, such as serogroup B, which accounts for 50% of meningococcal disease in developed countries.  Many commonly cited risk factors for meningococcal disease—such as hypogammaglobulinemia, systemic lupus erythematosus, functional hyposplenia, and asplenia—seem unlikely in this patient because she does not have a history of recurrent pyogenic infections, chronic medical conditions, or splenectomy. However, other risk factors, including human immunodeficiency virus (HIV) infection and complement deficiency, should be ruled out.  *Other Causes of Meningitis*  Although this patient’s illness is most consistent with a bacterial process, other causes of meningitis should be considered in the differential diagnosis. Enteroviral infection may be associated with meningitis, often with neutrophilic pleocytosis in the CSF during the early phase of the disease, but a concurrent rash in an adult would be unusual. Hemorrhagic viral diseases, including Ebola virus disease, are unlikely, because the patient had not recently traveled to an area where such diseases are endemic. Acute Epstein–Barr virus and cytomegalovirus infections are also unlikely, given the absence of elevated liver-enzyme levels and atypical lymphocytosis. Acute parvovirus infection with associated meningitis would be very unusual in the absence of arthropathy in an adult woman. Influenza virus infection can occasionally be associated with vasculopathy but is rarely associated with meningitis. The absence of respiratory symptoms and the negative rapid diagnostic test for influenza in this patient make this diagnosis unlikely.  Among noninfectious causes, systemic lupus erythematosus and drug-induced meningitis should be considered. Systemic lupus erythematosus may be associated with aseptic meningitis, but meningitis is rarely an initial manifestation of this condition, as would have been the case in this patient. Although ibuprofen is a common cause of drug-induced meningitis and is often associated with neutrophilic pleocytosis in the CSF, it was taken by this patient after the onset of her symptoms and is only rarely associated with petechiae.  *Recurrent Meningitis*  Why would this patient have an episode of meningitis as a child and again as an adult? A terminal complement deficiency involving C5, C6, C7, C8, or C9 components might explain not only her increased susceptibility to meningococcal disease but also the relatively mild nature of her illness (characterized by an absence of overt disseminated intravascular coagulation and purpura fulminans) and her rapid recovery. Furthermore, a deficiency in one of the terminal complement components, with the exception of C9, has been associated with recurrent meningococcal meningitis. Although the cause of this patient’s previous bout of bacterial meningitis, which occurred at 9 years of age, is not specified in the case history, her symptoms were reportedly similar to those of the current illness. Other causes of recurrent bacterial meningitis, such as anatomical defects with an associated CSF leak, seem unlikely, given that the patient had no reported history of otorrhea or rhinorrhea. Similarly, her reported good health during the 19 years after her first episode of bacterial meningitis makes chronic otitis media and sinusitis unlikely causes of her recurrent bacterial meningitis.  On the basis of this patient’s clinical and laboratory presentation and the epidemiology of each possible cause, meningococcemia with associated meningitis is the most likely diagnosis. I believe that the diagnostic test result was a positive blood culture for *N. meningitidis*. Furthermore, given the relatively mild and recurrent nature of the patient’s bacterial meningitis, I suspect that she has a hereditary terminal complement deficiency involving C5, C6, C7, or C8. I recommend testing for HIV antibodies. Additional diagnostic tests, which should be performed after the patient recovers from her illness, should include an assay for total hemolytic complement activity (*e.g.*, CH50) and, if that result is low, then testing for individual terminal complement components. If a diagnosis of complement deficiency is confirmed, testing should also be offered to the patient’s immediate relatives. Finally, a vaccine against *N. meningitidis* serogroup B has been recently approved and should be administered to this patient to help minimize her risk of future meningococcal disease.  *Dr. Virginia M. Pierce* (Pathology): Dr. Bachta, what was your impression when you evaluated this patient?  *Dr. Kelly Bachta* (Infectious Diseases): Our leading diagnosis was bacterial meningitis. Given the patient’s rash and her history of a previous episode of meningitis, we suspected recurrent *N. meningitidis* infection. The administration of intravenous vancomycin, ceftriaxone, and acyclovir had already been initiated and she had been given a dose of dexamethasone while she was in the emergency department, so we recommended that this regimen be continued as the evaluation proceeded. Through discussion with the state health department, we learned that the episode of meningitis that had occurred in the patient at 9 years of age had been due to *N. meningitidis* serogroup B. We strongly suspected a complement-related disorder and recommended consultation with an immunologist to help guide an evaluation for the presence of immunodeficiency. Because of the suspected *N. meningitidis* infection, we also recommended that the patient’s close contacts receive prophylaxis with oral rifampin. | Differential diagnoses:  Although the Gram stain strongly points to Neisseria meningitidis, other possible differentials based on similar presentations are:  1. Neisseria gonorrhoeae (gonococcemia): Can present with fever and rash, but classically causes tenosynovitis, dermatitis, and migratory polyarthritis rather than purpura fulminans.  2. Haemophilus influenzae (Gram-negative coccobacilli): May also cause meningitis, but morphology is different and usually seen in unvaccinated individuals.  3. Streptococcus pneumoniae (Gram-positive diplococci): Another leading cause of meningitis, but Gram stain morphology differs (Gram-positive, lancet-shaped). | The LLM lists only three “most likely other differential diagnoses”—Neisseria gonorrhoeae bacteremia, Haemophilus influenzae infection, and Streptococcus pneumoniae infection—and explains why the rash, fulminant course, and Gram’s stain morphology, respectively, do not fit perfectly. The overall focus on “a few common pathogens of bacterial meningitis or bacteremia”, without expansion of viral, rickettsial, or noninfectious causes, or discussion of other pathogens of septic vasopathy, is considerably less extensive than in the original article, and hence a score of 3 rather than higher. |
| Diagnostic accuracy | Neurologic form of immune reconstitution inflammatory syndrome (neuro-IRIS). | Epstein-Barr virus (EBV)-Associated CNS Infection (encephalitis or lymphoproliferative disorder).  Rationale:  1. EBV DNA in CSF: Direct evidence of EBV replication in the CNS.  2. Imaging Features: Progressive white matter abnormalities consistent with EBV-related demyelination or lymphocytic infiltration.  3. Clinical Context: HIV patient with immunosuppression (CD4 nadir 211) and prior Epstein-Barr virus (HSV)-2-associated Mollaret meningitis, suggesting susceptibility to herpesvirus reactivation.  4. Exclusion of Alternatives:  • tuberculous meningitis (TBM) lacks classic symptoms (fever, weight loss) and profound CSF hypoglycorrhachia.  • progressive multifocal leukoencephalopathy (PML) requires John Cunningham (JC) virus positivity.  • Human immunodeficiency virus encephalopathy (HIVE) unlikely with low CSF HIV RNA. | The LLM’s response is completely inconsistent with the original text. |
| Consistency of clinical management | Dr. Kelly Bachta (Infectious Diseases): Our leading diagnosis was bacterial meningitis. Given the patient’s rash and her history of a previous episode of meningitis, we suspected recurrent N. meningitidis infection. The administration of intravenous vancomycin, ceftriaxone, and acyclovir had already been initiated and she had been given a dose of dexamethasone while she was in the emergency department, so we recommended that this regimen be continued as the evaluation proceeded. Through discussion with the state health department, we learned that the episode of meningitis that had occurred in the patient at 9 years of age had been due to N. meningitidis serogroup B. We strongly suspected a complement-related disorder and recommended consultation with an immunologist to help guide an evaluation for the presence of immunodeficiency. Because of the suspected N. meningitidis infection, we also recommended that the patient’s close contacts receive prophylaxis with oral rifampin  Dr. Pierce: Dr. Bachta, would you tell us what happened next with this patient?  Dr. Bachta: Once we knew that the blood cultures were positive for N. meningitidis, we recommended a 7-day course of intravenous ceftriaxone. During this time, the patient defervesced and her rash resolved completely.  I saw her for a follow-up visit 2 weeks after discharge, at which time she felt well. We ad ministered the quadrivalent meningococcal conjugate vaccine, the 13-valent pneumococcal conjugate vaccine, and the H. influenzae type B vaccine. We also prescribed an oral antibiotic for emergency use in case she had recurring symptoms of meningitis at a time when she had limited access to medical care.  The patient was seen again 5 months later and received a second dose of quadrivalent meningococcal conjugate vaccine. By that time, she was known to have an isolated C8 deficiency.  The risk of N. meningitidis infection among persons with a terminal complement deficiency is 7000 to 10,000 times as high as the risk among persons without such a deficiency; furthermore, approximately 50% of persons with a terminal complement deficiency who have had N. meningitidis infection have recurrent infections. However, N. meningitidis infection is often milder and associated with lower mortality among persons  with a terminal complement deficiency than it is among persons without such a deficiency. We thought that this patient would benefit from immunization with the N. meningitidis serogroup B vaccine. It was unavailable in the United States at the time, but the patient successfully pursued vaccination in Canada. Finally, her immediate relatives were offered testing for complement deficiency, and her sister was also identified as having a C8 deficiency. | Management of this condition  Immediate management:  1. Empiric antibiotics (before culture results):  • High-dose intravenous ceftriaxone (or cefotaxime).  • Vancomycin and acyclovir may be added empirically until cultures clarify etiology (as was done).  • Once Neisseria meningitidis is confirmed, narrow to ceftriaxone alone.  2. Adjunctive therapy:  • Dexamethasone can be used early to reduce neurologic complications.  • Aggressive supportive care: intravenous (IV) fluids, monitoring for shock and disseminated intravascular coagulation (DIC).  3. Post-exposure prophylaxis: Close contacts require chemoprophylaxis with rifampin, ciprofloxacin, or ceftriaxone.  4. Address underlying risk:  • For this patient, C8 deficiency predisposes to recurrent meningococcal infections. She needs immunology follow-up, lifelong vigilance, and meningococcal vaccination (all available serogroups). | The management recommendations of LLM are highly consistent with the original article: empirical intravenous antibiotics (high-dose ceftriaxone, combined with vancomycin and acyclovir) should be started before the diagnosis is made, and ceftriaxone should be reduced to ceftriaxone alone when Neisseria meningitidis is confirmed, in combination with early dexamethasone, active fluid infusion and shock and disseminated intravascular coagulation monitoring. Prophylactic treatment with rifampicin, ciprofloxacin or ceftriaxone for close contacts, follow-up in the department of immunology, evaluation of complement deficiency and meningococcal vaccination are also recommended. The overall medication regimen and prevention and control ideas were almost completely consistent with the case report. |
| Completeness of pathological diagnosis | Dr. Diana Alame: We performed cultures of two sets of blood samples obtained in the emergency department before the administration of antibiotic agents. After approximately 24 hours of incubation in an automated blood-culture instrument, growth was detected in both aerobic bottles. Gram’s staining revealed intracellular and extra cellular gram-negative diplococci with a coffeebean shape (Fig. 3). The blood-culture broth was subcultured on solid mediums; after growth of bacterial colonies, the microorganism was identified as N. meningitidis by means of matrix-assisted laser desorption ionization–time-of-flight mass spectrometry. In vitro testing revealed susceptibility of the microorganism to both penicillin and ceftriaxone. The clinical microbiology laboratory also received a CSF specimen; Gram’s staining revealed numerous neutrophils, but no microorganisms were seen and the culture was negative.  The blood-culture isolate was submitted to the state health department laboratory for serotyping. Slide agglutination with pooled polyvalent and individual serogroup-specific antiserums to the capsular polysaccharides of 7 of 13 known N. meningitidis serogroups (A, B, C, Y, W-135, X, and Z) was performed. The isolate was non-groupable, indicating either that the isolate belonged to one of the serogroups of encapsulated meningococci that was not assayed or that the microorganism lacked a capsule. Noncapsular strains of N. meningitidis may be found as part of the nasopharyngeal flora but rarely cause invasive disease, since the capsule is an important virulence factor for the meningococcus. Further characterization of the isolate by means of realtime polymerase-chain-reaction testing at the Centers for Disease Control and Prevention revealed that the N. meningitidis isolate was non-groupable but had a capsule.  Since this was the patient’s second episode of invasive meningococcal disease, laboratory evaluation for immunodeficiency was performed. Titers of immunoglobulin G (IgG) antibodies against pneumococcal capsular polysaccharide and tetanus toxoid were measured and did not suggest abnormalities of either T-cell–independent or T-cell–dependent B-cell function. Levels of IgG, immunoglobulin A (IgA), and immunoglobulin M (IgM) antibodies were normal, as was the pattern of serum protein electrophoresis. However, assays for both total hemolytic complement activity (CH50) and alternative complement pathway activity (AH50) had abnormally low results; the CH50 level was less than 3 U per milliliter (reference range, 63 to 145), and the AH50 level was 12% of normal (reference range, 75 to 170). Levels of C3, C4, and factor B were normal. All three pathways of the complement system (*i.e.*, the classic pathway, the mannan-binding lectin pathway, and the alternative pathway) converge at the activation of C3 from their respective C3 convertases, and thus the observation of a low CH50 level in a patient with a normal C3 level indicates that the deficiency is localized downstream from this convergence point. The low AH50 level, together with the low CH50 level and the normal levels of C3, C4, and factor B, suggests a deficiency in one of the terminal complement components (C5, C6, C7, C8, or C9). Assessment of individual terminal complement components revealed a deficiency in C8 (C8 level, 13 U per milliliter; reference range, 33 to 58).  C8 deficiency confers a substantially elevated risk of invasive N. meningitidis infection. Although low-grade or transient meningococcal bacteremia can be overcome by opsonization and subsequent phagocytosis, higher-grade or persistent bacteremia requires the complement system for eradication. Vaccination is performed in an attempt to boost the early opsonophagocytic response. Ultimately, however, an intact and a sufficient response of the terminal complement components is crucial in overwhelming the defense mechanisms of this microorganism and in protecting against invasive disease. | • Examination: Petechiae and purpura are strongly suggestive of meningococcemia.  • Laboratory: Blood culture Gram stain shows Gram-negative, coffee-bean shaped diplococci, both intra- and extracellular. This is highly characteristic of Neisseria meningitidis.  • Predisposing factor: Complement deficiency (C8 deficiency) predisposes to recurrent Neisseria infections. | In his answer, LLM accurately described the feature of the Gram stain that “Gram-negative diplococci are located inside and outside polymorphonucleary leukocytes” and correctly interpreted it as typical of Neisseria meningitidis infection. C8 deficiency is an important susceptibility factor for recurrent Neisseria meningitidis infection. Immunological follow-up and complement examinations are necessary. Although the specificities of serogrouping and “unserogrouped but encapsulated” are not discussed, the etiology and key underlying diseases are fully covered at the clinically essential level. |
| Supplementary value | A 28-year-old woman was seen in the emergency department of this hospital because of headache, fever, and a rash.  The patient had been well until the morning of the day of admission, when she awoke with a severe generalized headache. The headache worsened with movement, was not relieved by acetaminophen or ibuprofen, and was accompanied by nausea and one episode of nonbloody, nonbilious emesis. The patient returned to sleep and awoke again several hours later with diffuse myalgias; her temperature was 37.7 °C. At that time, she also noted a rash on her abdomen, chest, and right arm; the lesions were small, red, flat, nonpruritic, and nonpainful. She described these symptoms to her father, a physician, who advised her to go to the emergency department of this hospital.  On arrival at the emergency department, the patient reported that the nausea had resolved and that mild neck stiffness had developed. She had had bacterial meningitis at 9 years of age, with symptoms similar to those of the current illness. She had no history of otitis media, sinusitis, pneumonia, or skin infections. She had a remote history of hospitalization after a motor vehicle accident. Ten months before the current illness, she had had an episode of nephrolithiasis with spontaneous passage of a renal stone. She also had a history of anxiety. Her medications were citalopram and a combined estrogen–progestin oral contraceptive. Immunizations were up to date and reportedly included vaccination against Neisseria meningitidis. She had no known allergies. She lived alone, worked as a lawyer, and had not recently traveled outside New England or had exposure to sick persons. She had a new boyfriend but was not currently sexually active. She did not smoke or use illicit drugs, and she rarely consumed alcohol. Her parents lived nearby; she had had contact with their two dogs but had not had any recent tick bites, mosquito bites, or exposure to other animals. There was no family history of immunodeficiency or autoimmune disease.  On examination, the patient was alert and oriented to person, place, and time. The temperature was 37.3 °C, the pulse 92 beats per minute, the blood pressure 122/68 mmHg, the respiratory rate 18 breaths per minute, and the oxygen saturation 100% while she was breathing ambient air. She had scattered petechiae on the chest, abdomen, and thighs and a purpuric lesion on the right shoulder that was tender on palpation without crepitus (Fig. 1). The remainder of the examination was normal. The hematocrit, hemoglobin level, red-cell indexes, prothrombin time, international normalized ratio, lactic acid level, and results of renal-function tests were normal, as were blood levels of calcium, total protein, albumin, globulin, alanine aminotransferase, aspartate aminotransferase, alkaline phosphatase, and lipase; other test results are shown in Table 1. Tests for influenza virus and respiratory syncytial virus were negative. Urinalysis revealed yellow, slightly cloudy urine, with trace ketones and 1+ bilirubin, occult blood, albumin, and urobilinogen by dipstick; there were 3 to 5 white cells and 5 to 10 red cells per high-power field, with 1+ bacteria, transitional cells, renal tubular cells, squamous cells, amorphous crystals, and mucin. Specimens of blood and urine were obtained for culture. Lorazepam was administered and a lumbar puncture was performed, after which ceftriaxone, vancomycin, acyclovir, and dexamethasone were administered intravenously. Results of the cerebrospinal fluid (CSF) analysis are shown in Table 1.  Within 90 minutes after the patient’s arrival at the emergency department, the temperature rose to 39.0 °C and the pulse increased to 122 beats per minute. Over the next 3 hours, additional petechiae were noted on her trunk, arms, and legs. Intravenous fluids, acetaminophen, ibuprofen, metoclopramide, potassium chloride, potassium phosphate, and sodium phosphate were administered. Additional laboratory tests were performed; the activated partial-thromboplastin time and levels of fibrinogen, haptoglobin, and lactate dehydrogenase were normal, and the d-dimer level was 785 ng per milliliter (reference range, <500). The patient was admitted to the hospital.  During the first hospital day, the patient continued to have a headache and was noted to have mild nuchal rigidity, but her fever and tachycardia resolved and her myalgias decreased; the remainder of the examination was unchanged. Additional laboratory tests were performed; test results are shown in Table 1. Ceftriaxone, vancomycin, acyclovir, and dexamethasone were continued. On the second hospital day, a diagnostic test result was received and additional diagnostic tests were performed.  Dr. Farrin A. Manian: This previously healthy 28-year-old woman presented with severe headache, nausea, vomiting, myalgias, and skin findings. The presence of an evolving petechial rash, with new lesions developing over a period of only a few hours, suggests a rapidly progressive disease requiring prompt recognition and management. A timeline of this patient’s presenta tion highlights the key features of this case (Fig. 2); these features were identified solely through history taking and physical examination, underscoring the crucial role of basic bedside clinical skills in the early detection of potential medical emergencies. In this patient, there appears to be a confluence of three disease processes causing a systemic illness with manifestations involving the skin and central nervous system. At the time of presentation to the emergency department, the patient’s heart rate was higher than 90 beats per minute and her white-cell count was greater than 12,000 per cubic millimeter. With the presence of these two findings, she met the criteria for the systemic inflammatory response syndrome (SIRS). Although thrombocytopenia was present, there was no evidence of overt disseminated intravascular coagulation; the d-dimer level was only mildly elevated and the fibrinogen level, prothrombin time, and activated partial-thromboplastin time were normal. Hypokalemia, hypomagnesemia, hypophosphatemia, and hyponatremia and the presence of albumin, red cells, and renal tubular cells in the urine of this previously healthy patient also raise the possibility of sepsis.  Vasculopathy  The second readily evident disease process in this patient revolves around her skin findings of petechiae and a purpuric lesion; these findings are suggestive of a vasculopathy. It is perhaps equally important to note the absence of bullae, gangrenous changes, and crepitance, findings that would suggest tissue necrosis or infarction, and the absence of generalized purpura, a finding that would be indicative of purpura fulminans. Vasculopathy in the presence of sepsis, or septic vasculopathy, often results in purpuric lesions and may be caused by a variety of organisms, including N. meningitidis, which accounts for approximately half the cases among nonimmune suppressed patients. Septic vasculopathy may occur within 24 hours after the onset of illness, as was observed in this patient, and biopsy of a lesion characteristically reveals intravascular fibrin thrombi of the superficial and deep-skin vessels, some with fibrinoid necrosis. These findings may be associated with bacterial endotoxemia.  Meningitis  In addition to SIRS and vasculopathy, this patient had acute meningitis, as indicated by the abrupt onset of severe headache and the neutrophilic pleocytosis in the CSF. The following clinical findings that are often associated with bacterial meningitis were noticeably absent in this case: mental-status changes, a CSF neutrophil count of greater than 1000 cells per cubic millimeter, visible organisms on Gram’s staining, hypoglycorrhachia (a low CSF glucose level), and an elevated CSF protein level. However, it is important to recognize that one or more clinical or laboratory signs or symptoms that are typical of meningitis—such as fever, nuchal rigidity, altered mental status, and a CSF white-cell count of greater than 1000 per cubic millimeter—may be absent in a substantial number of patients with this diagnosis,13 and the CSF glucose and protein levels may be normal. Similarly, of adults with meningococcal meningitis, impaired consciousness may be absent in 49% and Gram’s staining of CSF may be negative for organisms in 11%. The relatively mild CSF abnormalities in this patient may also indicate that she presented very early in the course of bacterial meningitis. Given the broad range of clinical and laboratory presentations of patients with bacterial meningitis and the likelihood of septic vasculopathy in this patient, a bacterial cause of meningitis remains the most likely diagnosis.  Bacterial Causes of Meningitis  Streptococcus pneumoniae should be a serious consideration in this patient because it is the most common cause of bacterial meningitis in adults, although it is not likely to be associated with vasculopathy. Haemophilus influenzae is a very uncommon cause of bacterial meningitis and vas culopathy in adults. Staphylococcus aureus can be associated with bacteremia and vasculopathy, but community-acquired meningitis and purpura are rarely caused by this organism. N. gonorrhoeae bacteremia can be associated with vasculopathy, but it would be unusual for this organism to cause meningitis; also, given the reported lack of sexual activity in this patient, this diagnosis is unlikely. Capnocytophaga canimorsus is a gramnegative bacillus associated with exposure to dogs. C. canimorsus infection may be manifested by sepsis, vasculopathy, and meningitis but often occurs in the presence of asplenia and dog bite; neither of these features was an apparent risk factor in this patient. Although Streptococcus pyogenes has been associated with meningitis and vasculopathy, group A streptococcal meningitis usually occurs in the presence of otitis media or sinusitis. Rickettsia rickettsii, the agent of Rocky Mountain spotted fever, can cause severe vasculopathy and occasionally meningitis; however, a rash developed within the first 24 hours after illness onset and the patient had no reported exposure to ticks, and thus an infection caused by this organism seems unlikely. Anthrax should be in the differential diagnosis of any illness with an abrupt onset that is characterized by sepsis and vasculopathy, but the absence of a fulminant disease course associated with shock and hemorrhagic meningitis makes this diagnosis very unlikely. Bacteremia due to Kingella kingae, a gram-negative bacillus, can be associated with signs and symptoms similar to those seen in this patient; however, K. kingae infection occurs primarily in children and is rarely complicated by meningitis. Other gram-negative bacilli, such as Pseudomonas aeruginosa, are also in the differential diagnosis; it is rare for P. aeruginosa to cause community-acquired meningitis, and when it does, it usually affects patients with underlying chronic medical conditions.  N. meningitidis is associated with a rapidly progressive, sometimes fulminant illness characterized by sepsis, petechial rash, purpura, and meningitis. This organism is the second most common cause of bacterial meningitis in adults, and most cases occur sporadically. Although the incidence of N. meningitidis infection is highest among children and young adults, the burden of disease is heaviest among adults 25 through 64 years of age; this patient is 28 years of age. Of adults with bacterial meningitis and concurrent rash, two thirds receive a diagnosis of meningococcal disease and most have a petechial rash. Of adults with meningococcal meningitis, 49% seek medical attention less than 24 hours after the onset of symptoms and 64% have a rash, of whom the majority have petechiae and one fourth have purpura, ecchymoses, or both; these features are consistent with this patient’s clinical presentation. Although this patient had received vaccination against N. meningitidis serogroups A, C, Y, and W-135, it is possible that she was infected with another serogroup, such as serogroup B, which accounts for 50% of meningococcal disease in developed countries.  Many commonly cited risk factors for meningococcal disease—such as hypogammaglobulinemia, systemic lupus erythematosus, functional hyposplenia, and asplenia—seem unlikely in this patient because she does not have a history of recurrent pyogenic infections, chronic medical conditions, or splenectomy. However, other risk factors, including human immunodeficiency virus (HIV) infection and complement deficiency, should be ruled out.  Other Causes of Meningitis  Although this patient’s illness is most consistent with a bacterial process, other causes of meningitis should be considered in the differential diagnosis. Enteroviral infection may be associated with meningitis, often with neutrophilic pleocytosis in the CSF during the early phase of the disease, but a concurrent rash in an adult would be unusual. Hemorrhagic viral diseases, including Ebola virus disease, are unlikely, because the patient had not recently traveled to an area where such diseases are endemic. Acute Epstein–Barr virus and cytomegalovirus infections are also unlikely, given the absence of elevated liver-enzyme levels and atypical lymphocytosis. Acute parvovirus infection with associated meningitis would be very unusual in the absence of arthropathy in an adult woman. Influenza virus infection can occasionally be associated with vasculopathy but is rarely associated with meningitis. The absence of respiratory symptoms and the negative rapid diagnostic test for influenza in this patient make this diagnosis unlikely.  Among noninfectious causes, systemic lupus erythematosus and drug-induced meningitis should be considered. Systemic lupus erythematosus may be associated with aseptic meningitis, but meningitis is rarely an initial manifestation of this condition, as would have been the case in this patient. Although ibuprofen is a common cause of drug-induced meningitis and is often associated with neutrophilic pleocytosis in the CSF, it was taken by this patient after the onset of her symptoms and is only rarely associated with petechiae.  Recurrent Meningitis  Why would this patient have an episode of meningitis as a child and again as an adult? A terminal complement deficiency involving C5, C6, C7, C8, or C9 components might explain not only her increased susceptibility to meningococcal disease but also the relatively mild nature of her illness (characterized by an absence of overt disseminated intravascular coagulation and purpura fulminans) and her rapid recovery. Furthermore, a deficiency in one of the terminal complement components, with the exception of C9, has been associated with recurrent meningococcal meningitis. Although the cause of this patient’s previous bout of bacterial meningitis, which occurred at 9 years of age, is not specified in the case history, her symptoms were reportedly similar to those of the current illness. Other causes of recurrent bacterial meningitis, such as anatomical defects with an associated CSF leak, seem unlikely, given that the patient had no reported history of otorrhea or rhinorrhea. Similarly, her reported good health during the 19 years after her first episode of bacterial meningitis makes chronic otitis media and sinusitis unlikely causes of her recurrent bacterial meningitis.  On the basis of this patient’s clinical and laboratory presentation and the epidemiology of each possible cause, meningococcemia with associated meningitis is the most likely diagnosis.  I believe that the diagnostic test result was a positive blood culture for N. meningitidis. Furthermore, given the relatively mild and recurrent nature of the patient’s bacterial meningitis, I suspect that she has a hereditary terminal complement deficiency involving C5, C6, C7, or C8. I recommend testing for HIV antibodies. Additional diagnostic tests, which should be performed after the patient recovers from her illness, should include an assay for total hemolytic complement activity (*e.g.*, CH50) and, if that result is low, then testing for individual terminal complement components. If a diagnosis of complement deficiency is confirmed, testing should also be offered to the patient’s immediate relatives. Finally, a vaccine against N. meningitidis serogroup B has been recently approved and should be administered to this patient to help minimize her risk of future meningococcal disease.  Dr. Virginia M. Pierce (Pathology): Dr. Bachta, what was your impression when you evaluated this patient?  Dr. Kelly Bachta (Infectious Diseases): Our leading diagnosis was bacterial meningitis. Given the patient’s rash and her history of a previous episode of meningitis, we suspected recurrent N. meningitidis infection. The administration of intravenous vancomycin, ceftriaxone, and acyclovir had already been initiated and she had been given a dose of dexamethasone while she was in the emergency department, so we recommended that this regimen be continued as the evaluation proceeded. Through discussion with the state health department, we learned that the episode of meningitis that had occurred in the patient at 9 years of age had been due to N. meningitidis serogroup B. We strongly suspected a complement-related disorder and recommended consultation with an immunologist to help guide an evaluation for the presence of immunodeficiency. Because of the suspected N. meningitidis infection, we also recommended that the patient’s close contacts receive prophylaxis with oral rifampin.  Dr. Diana Alame: We performed cultures of two sets of blood samples obtained in the emergency department before the administration of antibiotic agents. After approximately 24 hours of incubation in an automated blood-culture instrument, growth was detected in both aerobic bottles. Gram’s staining revealed intracellular and extra cellular gram-negative diplococci with a coffee-bean shape (Fig. 3). The blood-culture broth was subcultured on solid mediums; after growth of bacterial colonies, the microorganism was identified as N. meningitidis by means of matrix-assisted laser desorption ionization–time-of-flight mass spectrometry. In vitro testing revealed susceptibility of the microorganism to both penicillin and ceftriaxone. The clinical microbiology laboratory also received a CSF specimen; Gram’s staining revealed numerous neutrophils, but no microorganisms were seen and the culture was negative.  The blood-culture isolate was submitted to the state health department laboratory for serotyping. Slide agglutination with pooled polyvalent and individual serogroup-specific antiserums to the capsular polysaccharides of 7 of 13 known N. meningitidis serogroups (A, B, C, Y, W-135, X, and Z) was performed. The isolate was non-groupable, indicating either that the isolate belonged to one of the serogroups of encapsulated meningococci that was not assayed or that the microorganism lacked a capsule. Noncapsular strains of N. meningitidis may be found as part of the nasopharyngeal flora but rarely cause invasive disease, since the capsule is an important virulence factor for the meningococcus. Further characterization of the isolate by means of real-time polymerase-chain-reaction testing at the Centers for Disease Control and Prevention revealed that the N. meningitidis isolate was non-groupable but had a capsule.  Since this was the patient’s second episode of invasive meningococcal disease, laboratory evaluation for immunodeficiency was performed. Titers of IgG antibodies against pneumococcal capsular polysaccharide and tetanus toxoid were measured and did not suggest abnormalities of either T-cell–independent or T-cell–dependent B-cell function. Levels of IgG, IgA, and IgM antibodies were normal, as was the pattern of serum protein electrophoresis. However, assays for both total hemolytic complement activity (CH50) and alternative complement pathway activity (AH50) had abnormally low results; the CH50 level was less than 3 U per milliliter (reference range, 63 to 145), and the AH50 level was 12% of normal (reference range, 75 to 170). Levels of C3, C4, and factor B were normal. All three pathways of the complement system (*i.e.*, the classic pathway, the mannan-binding lectin pathway, and the alternative pathway) converge at the activation of C3 from their respective C3 convertases, and thus the observation of a low CH50 level in a patient with a normal C3 level indicates that the deficiency is localized downstream from this convergence point. The low AH50 level, together with the low CH50 level and the normal levels of C3, C4, and factor B, suggests a deficiency in one of the terminal complement components (C5, C6, C7, C8, or C9). Assessment of individual terminal complement components revealed a deficiency in C8 (C8 level, 13 U per milliliter; reference range, 33 to 58).  C8 deficiency confers a substantially elevated risk of invasive N. meningitidis infection. Although low-grade or transient meningococcal bacteremia can be overcome by opsonization and subsequent phagocytosis, higher-grade or persistent bacteremia requires the complement system for eradication. Vaccination is performed in an attempt to boost the early opsonophagocytic response. Ultimately, however, an intact and a sufficient response of the terminal complement components is crucial in overwhelming the defense mechanisms of this microorganism and in protecting against invasive disease.  Dr. Pierce: Dr. Bachta, would you tell us what happened next with this patient?  Dr. Bachta: Once we knew that the blood cultures were positive for N. meningitidis, we recommended a 7-day course of intravenous ceftriaxone. During this time, the patient defervesced and her rash resolved completely. ministered the quadrivalent meningococcal conjugate vaccine, the 13-valent pneumococcal conjugate vaccine, and the H. influenzae type B vaccine. We also prescribed an oral antibiotic for emergency use in case she had recurring symptoms of meningitis at a time when she had limited access to medical care.  The patient was seen again 5 months later and received a second dose of quadrivalent meningococcal conjugate vaccine. By that time, she was known to have an isolated C8 deficiency.  The risk of N. meningitidis infection among persons with a terminal complement deficiency is 7000 to 10,000 times as high as the risk among persons without such a deficiency; furthermore, approximately 50% of persons with a terminal complement deficiency who have had N. meningitidis infection have recurrent infections. However, N. meningitidis infection is often milder and associated with lower mortality among persons with a terminal complement deficiency than it is among persons without such a deficiency. We thought that this patient would benefit from immunization with the N. meningitidis serogroup B vaccine. It was unavailable in the United States at the time, but the patient successfully pursued vaccination in Canada. Finally, her immediate relatives were offered testing for complement deficiency, and her sister was also identified as having a C8 deficiency. | Here is a structured answer based on the case description and the Gram stain image:  1. Reasoning steps and 3 most likely differential diagnoses  Reasoning steps:  • Symptoms: The 28-year-old woman presented with acute onset of fever, severe headache, myalgias, petechial rash, mild neck stiffness, and rapid clinical progression.  • Examination: Petechiae and purpura are strongly suggestive of meningococcemia.  • Laboratory: Blood culture Gram stain shows Gram-negative, coffee-bean shaped diplococci, both intra- and extracellular. This is highly characteristic of Neisseria meningitidis.  • Predisposing factor: Complement deficiency (C8 deficiency) predisposes to recurrent Neisseria infections.  Key findings leading to diagnosis:  • Acute febrile illness with meningismus  • Petechial and purpuric rash (suggestive of meningococcemia)  • Gram stain: Gram-negative diplococci inside neutrophils  • Blood culture positivity  Differential diagnoses:  Although the Gram stain strongly points to Neisseria meningitidis, other possible differentials based on similar presentations are:  1. Neisseria gonorrhoeae (gonococcemia): Can present with fever and rash, but classically causes tenosynovitis, dermatitis, and migratory polyarthritis rather than purpura fulminans.  2. Haemophilus influenzae (Gram-negative coccobacilli): May also cause meningitis, but morphology is different and usually seen in unvaccinated individuals.  3. Streptococcus pneumoniae (Gram-positive diplococci): Another leading cause of meningitis, but Gram stain morphology differs (Gram-positive, lancet-shaped).  Most likely diagnosis  Invasive meningococcal disease (meningococcemia with meningitis) due to Neisseria meningitidis.  This is supported by:  • Rapid onset of symptoms  • Petechial/purpuric rash  • Gram-negative diplococci  • Complement deficiency (C8) as an underlying risk factor.  Management of this condition  Immediate management:  1. Empiric antibiotics (before culture results):  • High-dose intravenous ceftriaxone (or cefotaxime).  • Vancomycin and acyclovir may be added empirically until cultures clarify etiology (as was done).  • Once Neisseria meningitidis is confirmed, narrow to ceftriaxone alone.  2. Adjunctive therapy:  • Dexamethasone can be used early to reduce neurologic complications.  • Aggressive supportive care: IV fluids, monitoring for shock and DIC.  3. Post-exposure prophylaxis: Close contacts require chemoprophylaxis with rifampin, ciprofloxacin, or ceftriaxone.  4. Address underlying risk:  • For this patient, C8 deficiency predisposes to recurrent meningococcal infections. She needs immunology follow-up, lifelong vigilance, and meningococcal vaccination (all available serogroups).  References:  • Mandell, Douglas, and Bennett’s Principles and Practice of Infectious Diseases.  • Harrison’s Principles of Internal Medicine. | The answers of LLM were mainly a structured summary of the key points of the case, a restatement of the relationship between symptoms, signs, Gram’s stain and complement deficiency and Neisseria meningitidis infection, and standard textbook treatment recommendations and two reference books. Compared with the original text, it introduces few new guideline points, risk assessment tools, or additional pathophysiological details, and is more “condensed and reorganized information” than obvious value-added, so the supplementary value is only rated as 2 points. |

LLM: large language model.
